# Distribution of corpora amylacea in the human midbrain: using synchrotron radiation phase-contrast microtomography, high-field magnetic resonance imaging, and histology

**DOI:** 10.3389/fnins.2023.1236876

**Published:** 2023-10-04

**Authors:** Ju Young Lee, Andreas F. Mack, Ulrich Mattheus, Sandro Donato, Renata Longo, Giuliana Tromba, Thomas Shiozawa, Klaus Scheffler, Gisela E. Hagberg

**Affiliations:** ^1^Graduate Training Centre of Neuroscience, Eberhard Karl's University of Tübingen, Tübingen, Germany; ^2^High Field Magnetic Resonance, Max Planck Institute for Biological Cybernetics, Tübingen, Germany; ^3^Institute of Clinical Anatomy and Cell Analysis, Eberhard Karl's University of Tübingen, Tübingen, Germany; ^4^Department of Physics and STAR-LAB, University of Calabria, Rende, Italy; ^5^Division of Frascati, Istituto Nazionale di Fisica Nucleare (INFN), Frascati, Italy; ^6^Department of Physics, University of Trieste, Trieste, Italy; ^7^Division of Trieste, Istituto Nazionale di Fisica Nucleare (INFN), Trieste, Italy; ^8^Elettra—Sincrotrone Trieste S.C.p.A, Basovizza, Italy; ^9^Department of Biomedical Magnetic Resonance, University Hospital Tübingen, Tübingen, Germany

**Keywords:** corpora amylacea, phase-contrast X-ray microtomography, human brainstem, high-field magnetic resonance imaging, histology

## Abstract

Corpora amylacea (CA) are polyglucosan aggregated granules that accumulate in the human body throughout aging. In the cerebrum, CA have been found in proximity to ventricular walls, pial surfaces, and blood vessels. However, studies showing their three-dimensional spatial distribution are sparse. In this study, volumetric images of four human brain stems were obtained with MRI and phase-contrast X-ray microtomography, followed up by Periodic acid Schiff stain for validation. CA appeared as hyperintense spheroid structures with diameters up to 30 μm. An automatic pipeline was developed to segment the CA, and the spatial distribution of over 200,000 individual corpora amylacea could be investigated. A threefold—or higher—density of CA was detected in the dorsomedial column of the periaqueductal gray (860–4,200 CA count/mm^3^) than in the superior colliculus (150–340 CA count/mm^3^). We estimated that about 2% of the CA were located in the immediate vicinity of the vessels or in the peri-vascular space. While CA in the ependymal lining of the cerebral aqueduct was rare, the sub-pial tissue of the anterior and posterior midbrain contained several CA. In the sample with the highest CA density, quantitative maps obtained with MRI revealed high R2^*^ values and a diamagnetic shift in a region which spatially coincided with the CA dense region.

## 1. Introduction

Corpora amylacea (CA) are polyglucosan aggregates that tend to accumulate in several organs throughout aging (Cavanagh, 1999; Pirici and Margaritescu, 2014; Riba et al., 2021). In the human central nervous system, these granules are found close to ventricular walls, pial surfaces, and blood vessels. At times they have been observed enclosed within cells, such as astrocytes (Palmucci et al., 1982; Navarro et al., 2018; Augé et al., 2019) or neurons (Anzil, 1974; Ikeda et al., 1987; Yoshikawa et al., 1990). However, they have mainly been observed outside the cells in the extracellular matrix (Navarro et al., 2018). The exact mechanism of their formation is not yet fully understood. Generally, investigating CA can help to elucidate their role in brain tissue and may give insights into the process of brain aging. CA are usually detected in histology sections, using the periodic acid Schiff staining technique. There is currently a paucity of reports describing the spatial distribution of CA in three dimensional space.

Growing evidence suggests that CA are waste containers. They include ubiquitin and p62 proteins, which are both involved in waste removal processes (Augé et al., 2018). Immunohistochemistry has shown that CA include neo-epitopes that are recognized by endogenous immunoglobins (Augé et al., 2019). Several studies have demonstrated phagocytosis of CA by macrophages (Suzuki et al., 2012; Ohara et al., 2019; Riba et al., 2019). Furthermore, proteins of fungal and bacterial origin have been found within cerebral CA (Pisa et al., 2016; Zhan et al., 2021).

The ultrastructure of CA has been studied with electron microscopy (Ikeda et al., 1987; Leel-Ossy, 2001; Augé et al., 2019). CA are densely packed with fibril-like structures that are randomly oriented. Intracellular CA are smaller (<10 μm diameter) and less densely packed with fibril-like structures and are surrounded by a membrane; extracellular CA are bigger (up to 30 μm diameter), compactly packed, and do not have a membrane. Cellular organelles such as mitochondria are sometimes found on the edges of CA (Navarro et al., 2018)

CA may play an important role in neurodegeneration and vascular disease (Kosaka et al., 1981; Rohn, 2015). Recently, it was found that CA are altered in the dental gyrus of the hippocampus of patients with Alzheimer's disease compared to healthy volunteers. CA density undergoes changes during the disease progression, possibly reflecting defensive waste-removal mechanisms that are insufficient at the early stage, but which tend to increase at the intermediate stages and eventually fail in the last stages of the disease (Wander et al., 2022). Several clinical cases also show potential association of CA and medial temporal lobe epilepsy (Choi et al., 2003; Das et al., 2011).

There are still many open questions concerning CA. One such question is related to their detectability within the human brain. Using magnetic resonance imaging (MRI), CA-dependent alterations of the T1 and T2 weighted signals have been reported *in vivo* (Abel et al., 2010; Lee et al., 2017; Pimentel et al., 2020). Quantitative MRI of *ex vivo* tissue samples is an emerging technique and can potentially reveal signal alterations linked with CA. For instance, quantitative susceptibility mapping (QSM) and the effective transversal relaxation rate (R2^*^) have been used to measure calcifications and iron content, and high resolution QSM has been used to detect paramagnetic shifts likely originating from iron-binding beta amyloid plaques in Alzheimer's disease (Tuzzi et al., 2020). In CA, tissue components like calcium, iron, and copper have been observed with X-ray microanalysis (Singhrao et al., 1993; Tokutake et al., 1995), which could lead to local alterations of the magnetic field detectable with gradient-echo MRI techniques.

In the present study, we used three techniques to study the CA in human brain stems. First, gradient echo images were acquired using a high field MRI scanner to visualize the anatomy of the samples prior to paraffin embedding and to obtain quantitative MRI parameters. To be able to resolve individual CA which have diameters <30 μm (Pirici and Margaritescu, 2014), we used synchrotron radiation phase-contrast X-ray microtomography (SR PhC-μCT) to acquire three-dimensional images with voxel sizes of 1 × 1 × 1 μm^3^ and 5 × 5 × 5 μm^3^. Finally, the specimens were prepared for histology to verify the presence of CA. By using automated segmentation on SR PhC-μCT images with 1 μm isotropic voxels, we could identify a large number of CA in the midbrain and investigate their spatial distribution. A higher number of CA were found along the midline of the mesencephalon, corresponding to the dorsomedial periacqueductal gray (dmPAG). MRI of the sample with the highest CA density showed increased R2^*^ and more diamagnetic QSM values in the dmPAG area than in the surrounding tissue. The proposed methodology opens up novel prospects for studying CA in *ex vivo* brain tissue.

## 2. Materials and methods

### 2.1. Sample preparation 1

*Postmortem* human brain stem samples (N = 4, Age = 75.75 ± 5.21, 3 females/1 male) were collected through the body donor program at the Institute of Clinical Anatomy and Cell Analysis, Department of Anatomy, Eberhard Karls University of Tübingen. The donors provided their informed consent in line with the declaration of Helsinki for research purposes. The ethics commission at the Medical Department of the University of Tübingen approved the procedure. The donors had no history of neurological disease. The samples were fixed in formaldehyde solution (Roti^®^-Histofix 4 % phosphate-buffered formaldehyde solution, pH 7 from Carl Roth GmbH + Co. KG, Karlsruhe, Germany; 140 mM NaCl and 2.7 mM KCl from Sigma-Aldrich Chemie GmbH, Merck KG, Taufkirchen, Germany) for a minimum of 4 weeks and kept at room temperature throughout this study (Nazemorroaya et al., 2022).

### 2.2. High-field magnetic resonance imaging

To avoid any background signal, the tissue was immersed in a FC-770 Fluorinert solution (Sigma-Aldrich Chemie GmbH, Taufkirchen, Germany) during the MRI acquisition. Multi-echo gradient echo images were acquired at 14.1T (Bruker Biospec, Ettlingen, Germany). The samples were positioned at the center of the magnet and the radio-frequency (RF) coil followed by global shimming, frequency adjustment, reference power optimization, and receiver gain adjustment using the automatic set-up method provided by the manufacturer (software: Paravision 6.0.1). We used a BFG240-120-S14 gradient system and an in-house built quadrature volume transceiver RF coil. Scanning parameters are provided in Supplementary Table 1.

Maps of the effective transverse relaxation rate (R2^*^) were generated by fitting a mono-exponential decay curve to the square of the magnitude using least square procedure. Quantitative susceptibility mapping image was calculated from the first echo after ROMEO unwrapping (Dymerska et al., 2021), and RESHARP background removal using Tikhonov regularization with a factor of −3 and a kernel size of twice the in-plane voxel size (Sun and Wilman, 2014), prior to dipole inversion with the iLSQR algorithm in STI Suite v3.0 (Liu, 2017).

### 2.3. Sample preparation 2

After acquisition of MRI images, the brainstem specimens were cut into 1 cm thick blocks perpendicularly to the rostro-caudal (superior-inferior) axis, dehydrated in increasing ethanol concentration, transferred to Xylene, and embedded in paraffin for SR PhC-μCT. Data acquired from the paraffin blocks covering the midbrains are presented here. For sample 1, 3, and 4, two blocks were measured, where one included the midbrain section covering the superior colliculus and the other included the inferior colliculus. For sample 2, the midbrain was smaller and a single block covered the entire midbrain.

### 2.4. Synchrotron radiation imaging

The tissue samples were scanned using synchrotron-radiation X-rays at the Elettra Synchrotron facility [SYRMEP beamline (Dullin et al., 2021)] across three beamtimes between 2019 and 2021 (details in Supplementary Table 2). The Elettra ring operated in Top-Up mode at an electron energy of 2.0 GeV. The polychromatic beam passed through 1 mm silicon filter, yielding the mean beam energy of 20.7 keV (mode = 19.6 keV) and a flux of 1.1 × 10^11^ photon/s/mm^2^ during the first two beam times, and about 24% lower flux during the third. Using a half-acquisition mode (Wang, 2002), 3600 projections distributed around 360° were acquired with 200 ms exposure time by a water-cooled 16-bits CMOS camera (Hamamatsu C11440-22C-Flash4.0 v2). The propagation-based phase-contrast imaging technique was used with two settings (Brombal et al., 2018; Donato et al., 2022): 900 mm sample-to-detector distance for the 5 μm isotropic voxel images and 200 mm sample-to-detector distance for the 1 μm isotropic voxel images. Thanks to this geometry and to the spatial coherence of the synchrotron source, the employed technique enables us to exploit phase-contrast effects that result in an edge enhancement arising at the boundaries between tissue components having different compositions (Brombal, 2020).

We used the SYRMEP Tomo Project software suite for microtomography reconstruction (Brun et al., 2015). The projections were processed with conventional flat-fielding and Paganin's phase retrieval algorithm (Paganin et al., 2002). The latter was used with a delta/beta ratio of 50 for the 5 μm isotropic voxel images and 20 for the 1 μm isotropic voxel images. Three-dimensional images were reconstructed using filtered back projection.

### 2.5. Histology

Histology was conducted on the paraffin blocks that covered the superior colliculus. The paraffin embedded blocks were manually cut at 10 μm using a rotational microtome (Leica, Wetzlar, Germany). For subjects 1, 3, and 4, every 10th section was stained with the luxol fast blue—cresyl violet method, and for subject 2, serial sections were stained with luxol fast blue—cresyl violet. Furthermore, selected slides were stained using periodic-acid Schiff–hematoxylin method for subject 1, 3, and 4 and additional hematoxylin and eosin stain for subject 4. Stained slices were digitized using a Zeiss Axio scan microscope (Zeiss, Jena, Germany).

### 2.6. CA segmentation from high resolution phase contrast microtomography

We used ImageJ software (Schindelin et al., 2012; Schneider et al., 2012) for segmentation of individual CA granules in the 1 μm isotropic voxel images.

The images were first downscaled by a factor of 0.5 using trilinear interpolation, resulting in 2 μm isotropic voxel size, which was sufficient for our purpose. Then the background noise was removed using second degree polynomial fitting to each slice with Xlib plugin (Münch, 2021).

Next, we used a hysteresis threshold for identifying hyperintense objects using 3D ImageJ Suite plugin (Ollion et al., 2013). Hysteresis thresholding was applied with [mode + 6^*^standard deviation] and [mode + 4.5^*^standard deviation] as high and low thresholds respectively. Using the MorpholibJ package (Legland et al., 2016), two morphological features were calculated for each segmented object: volume and sphericity. The sphericity was computed as 36^*^π^*^V^2^/S^3^, where V is the volume and S is the surface area of the object. The connected components with a volume larger than 55 voxels (365 μm^3^) and a sphericity >0.85 (Supplementary Figure 1) were identified as CA. For each segmented CA, the diameter was calculated as (6V/π)^1/3^.

The minimum diameter of detected CA was set to 8.87 μm, as determined by the volume cut-off. Based on prior research on CA using electron microscopy, the estimated diameter of CA distribution is 5.66 ± 3.94 μm (Navarro et al., 2018). This is a suitable range for electron microscopy that has voxel sizes in the nanometer range and can actually visualize the fibril-like structures within the CA. Even with the sub-micron resolution, optical microscopy and the SR PhC-μCT method rely heavily on the circular morphology of the granule for identifying CA. In histology, which is used in this study to validate SR PhC-μCT, CA are usually investigated in a size range above 10 μm. Walls of the capillary blood vessel also shows periodic-acid Schiff reaction and, depending on the sectioning orientation of the histological slide, it can be difficult to dissociate CA and obliquely cut fragments of the capillary vessel wall. Furthermore, oligodendrocytes, which can have diameters of up to 8 μm, also appear as spherical shapes with hyperintense signals in SR PhC-μCT. Therefore, we chose a volume cut off close to 10 μm in this study, which is above the typical range observed for oligodendrocytes and makes inclusion of vessel fragments unlikely.

### 2.7. Density map

3D density maps were generated with the 3D ImageJ Suite plugin (Ollion et al., 2013) using “3D density” function (Boudier, 2023). In this study, the density map is used for visualization purposes only. The density metric is a sum of Gaussian weighted distance between neighboring granules. For each coordinate, we calculated


$$\begin{array}{l} \sum \left(e^{-dist_{i}^{2*}C}\right) \end{array}$$

where *dist*_*i*_ is the Euclidean distance to the ith closest neighboring granule, which was set to 5. C=1/(2^*^σ^2^) where σ is the standard deviation of the Gaussian function, which we set to 50 μm. For each coordinate, Gaussian weights for the five nearest neighboring CA were added. The unit for the resulting metric is arbitrary. Different pairs of parameters are known to lead to similar density maps (Zakirov et al., 2021)

### 2.8. Region-of-interest analysis

ITK-SNAP software (Yushkevich et al., 2006) was used to obtain a binary map for the tissue masks. To identify the dorsomedial periaqueduct region, we referred to the human brain stem atlas (Paxinos et al., 2020). The mid-line of the brain stem was used as a reference. We took this reference as the center line and selected a region with a 1.6 mm width. For the block containing the inferior colliculus, the dorsomedial periaqueductal gray, which extended to the pial surface, was included in the analysis. For the superior colliculus, the entire tissue within the measured field-of-view was included (Supplementary Figure 2).

In subject 2, SR PhC-μCT of 1 μm isotropic voxel size images covering dmPAG was not acquired. This is because dmPAG was not selected as a target area in the original plan for the measurement at the synchrotron facility.

### 2.9. Vessel segmentation

For SR PhC-μCT data, the vessels were segmented as described in our prior work (Lee et al., 2022) with additional steps to separate the CA from the vessels. For that purpose, the voxel intensity in locations with segmented CA was replaced by the mode value of the voxels identified as tissue. Then the 3D median filter was applied, followed by Deriche-Canny edge detection, hysteresis thresholding, and post-processing as described in Lee et al. (2022). By doing so, we could extract the correct vessel-tissue boundary (Supplementary Figure 3). With the resulting vessel binary map and CA binary map, we computed the overlap in order to determine the percentage of CA in the immediate vicinity or within the perivascular space. For subject 4, larger clusters of CA were present in dmPAG, so this approach could not be performed on the image covering the region of subject 4.

For the microscopy images, vessels were semi-automatically segmented based on thresholding with Qupath software (Bankhead et al., 2017). Since the perivascular space is void of tissue, thus showing a white background color, any connected region with values above 205 (RGB channel average) and areas larger than 5,000 μm^2^ were selected as candidates. In order to include blood vessels filled with red blood cells, connected regions with a red channel value below 5 and area larger than 1,000 μm^2^ were also selected as candidates. Then the regions that corresponded to the cerebral aqueduct, tissue folding, or tissue tears were manually deleted. Each remaining vessel segment was visually inspected for CA.

## 3. Results

### 3.1. Corpora amylacea show hyperintensity in phase-contrast X-ray microtomography

**Figure 1:**
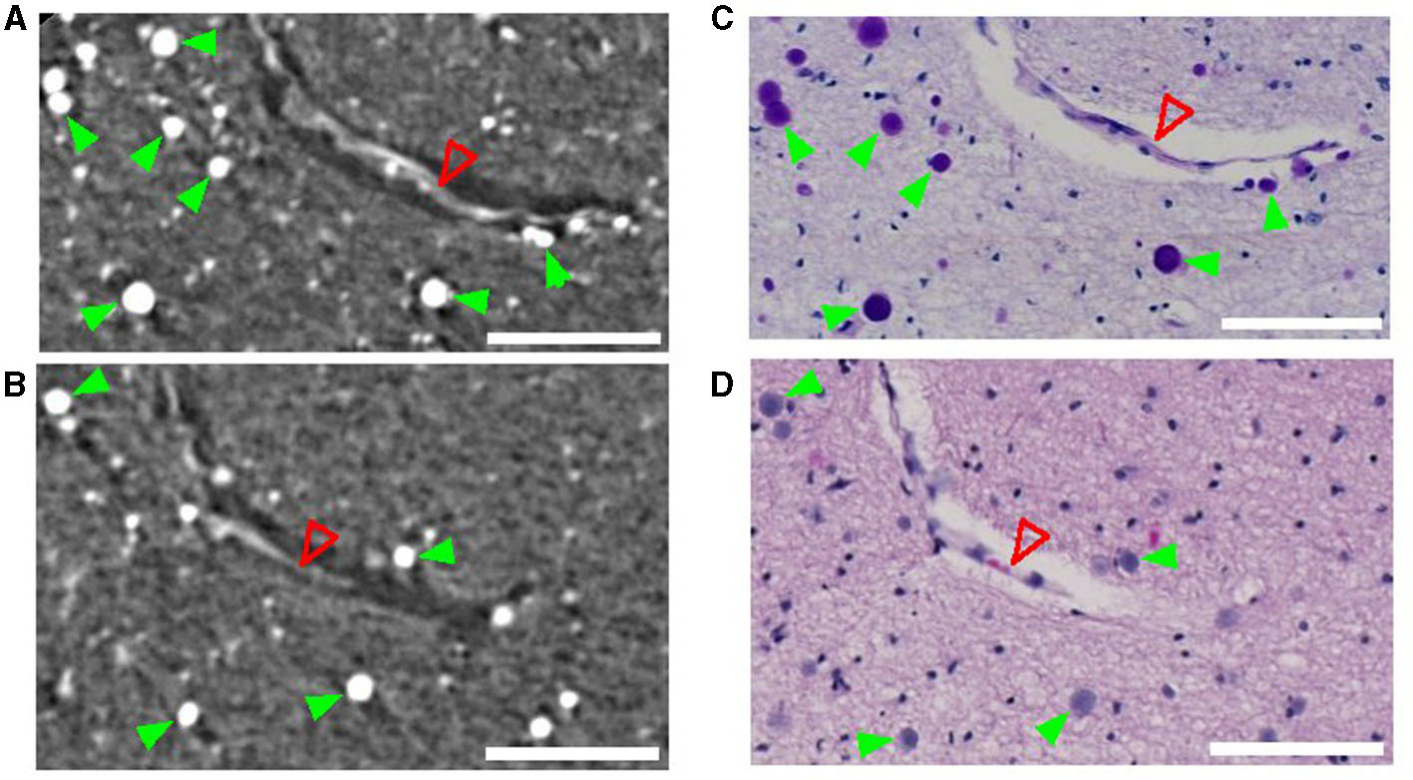
Corpora amylacea in synchrotron radiation phase-contrast microtomography (SR PhC-μCT) and histological section. SR PhC-μCT showing several corpora amylacea **(A, B)** and matching region stained with the periodic acid-Shiff method **(C)** and the hematoxylin and eosin stain **(D)**. Green filled arrowheads point to corpora amylacea and the open red arrowhead point to a collapsed blood vessel. Scale bars = 100 μm. **(A, B)** are maximum intensity projection across 10 μm in order to match the slice thickness of the histology sections **(C, D)**.

CA are densely packed granules that can be detected using SR PhC-μCT. They are characterized by their hyperintensity and spherical shape (Hieber et al., 2016). As shown in Figures 1A, B, individual CA stand out and can be identified by visual inspection in high-resolution SR PhC-μCT images. By comparing these images with histology sections from the matching regions, the exact granules found in SR PhC-μCT were located and verified as CA. In sections processed with the periodic acid Schiff reaction, CA appeared as dark purple structures (Figure 1C), while with the hematoxylin and eosin method, CA displayed a light purple color (Figure 1D).

Using automated segmentation, more than 2^*^10^5^ CA could be segmented from the high-resolution SR PhC-μCT from four brain stems (Supplementary Video 1, Supplementary Table 3). To the best of our knowledge, the number of CA reported so far in a single study has been only up to several hundred for electron microscopy studies (Navarro et al., 2018), and several thousand for microscopy studies (Xu et al., 2021).

### 3.2. The dorsomedial periacqueductal gray is rich with corpora amylacea

**Figure 2:**
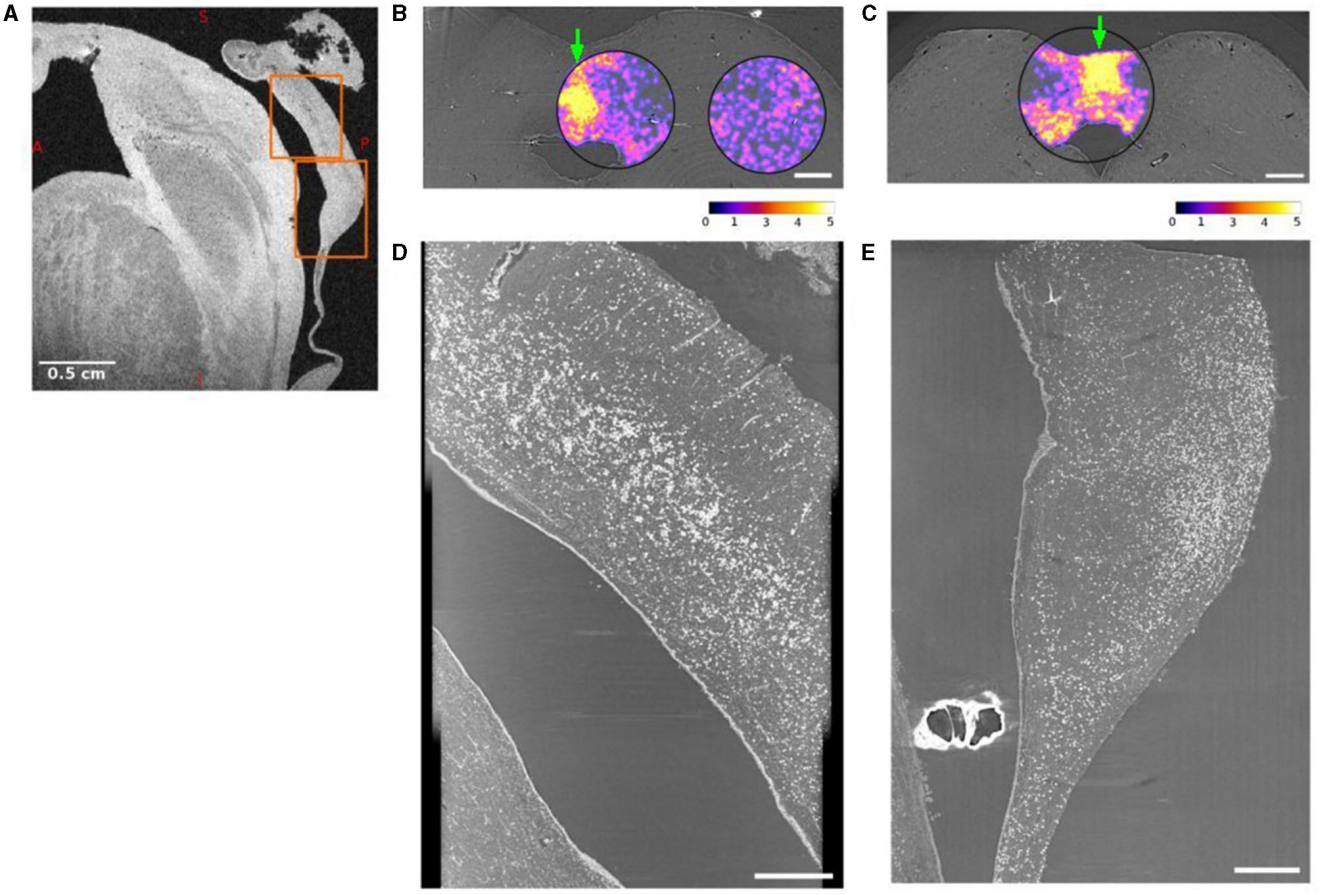
Dorsomedial periaqueductal gray (dmPAG) has abundant corpora amylacea. **(A)** Sagittal view of the midbrain from a T2* weighted magnetic resonance imaging (MRI). **(B, C)** Axial views of synchrotron radiation phase-contrast microtomography (SR PhC-μCT) acquired with 5 μm isotropic voxel size from the midbrain of subject 4. The density of corpora amylacea in the superior **(B)** and inferior **(C)** midbrain are shown as violet/yellow maps. The density maps were obtained from 1 μm isotropic voxel size SR PhC-μCT. The density of corpora amylacea is clearly higher in the dmPAG region than in the collicular regions. The abundance of corpora amylacea can also be appreciated in the sagittal maximum-intensity projections across the dmPAG at the levels of the superior **(D)** and inferior **(E)** colliculi. The regions used in **(D, E)** are indicated with arrows in panel **(B, C)** respectively. The projections are across 60 μm, matching the voxel size in MRI shown in **(A)**, where the same regions are indicated with orange rectangles. Scale bars **(A)** = 0.5 cm, **(B, C)** = 1 mm, **(D, E)** = 0.5 mm. Calibration bar shows the arbitrary unit for density calculation.

A large number of CA were found in the dmPAG area in all subjects (Figure 2, Supplementary Figures 4–6). Figure 2 demonstrates the most extreme case with the highest number of CA found in subject 4. Figure 2A shows a sagittal view of an MRI image with an orange box as overlay to show the field-of-view used for SR PhC-μCT. Two axial views of the midbrain at the level of the superior colliculus and inferior colliculus are shown in Figures 2B, C, respectively. The density map of CA is overlayed, demonstrating a high density in dmPAG. The sagittal view of the superior midbrain shows a high density of CA along a line running parallel with the cerebral aqueduct (Figure 2D). In the inferior midbrain, CA were also found in dmPAG (Figure 2E).

**Figure 3:**
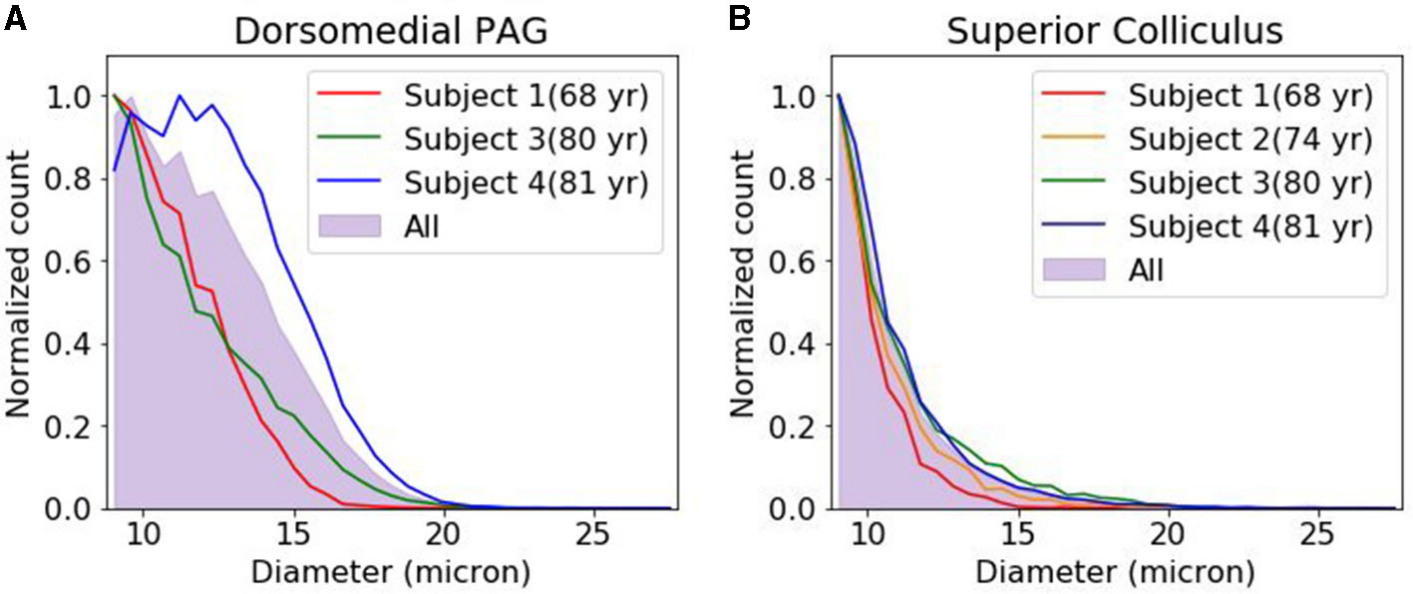
Corpora amylacea size distribution. The distribution of corpora amylacea diameter in the dorsomedial periaqueductal gray **(A)** and collicular **(B)** regions. Each histogram was normalized by the bin with the largest count. For subject 2, images were acquired from superior colliculus region but not from dorsomedial periacqueductal gray. PAG, periacqueductal gray.

We compared the density and size of the CA found in the dmPAG region with those found in the adjacent collicular regions. The dmPAG showed a higher density of CA compared to the collicular region, showing at least 3-fold more CA (Table 1). The size of the CA was also larger in the dmPAG region compared to the colliculus region (Figure 3)

**Table 1:** Corpora amylacea density in the dorsomedial periacqueductal gray and the superior colliculus region.

**Brain stem region**	**Subject**	**Corpora amylacea density (count/mm^3^)**
Dorsomedial periaqueductal gray	Subject 1 (68yr)	855
	Subject 3 (80yr)	1020
	Subject 4 (81yr)	4197
	mean ± std	**2,035** **±1538**
Superior colliculus	Subject 1 (68yr)	150
	Subject 2 (74yr)	217
	Subject 3 (80yr)	340
	Subject 4 (81yr)	315
	mean ± std	**226** **±76**

The difference between corpora amylacea density in the two regions ranged from 3 folds (subject 3) to 13 folds (subject 4). For subject 2, images were acquired from the superior colliculus region but not from dorsomedial periacqueductal gray. Values were in bold to highlight the ‘mean ± std' values.

In two subjects, the paraffin blocks from the lower brainstem included more caudal regions, corresponding to the transition zone between the inferior collicular commissure to the superior medullary velum (Supplementary Figure 7). These regions were also highly populated with CA.

### 3.3. Cerebral spinal fluid and corpora amylacea

Prior studies report finding abundant CA in the vicinity of cerebral spinal fluid (CSF)-filled spaces, such as the ependymal or subpial regions (Alder, 1953; Sakai et al., 1969; Nam et al., 2012). Since the periventricular and subpial region were not entirely scanned with SR PhC-μCT, we used microscopic images of the periodic acid Shiff-stained sections and luxol fast blue—cresyl violet-stained sections to observe the distribution of CA in these regions.

**Figure 4:**
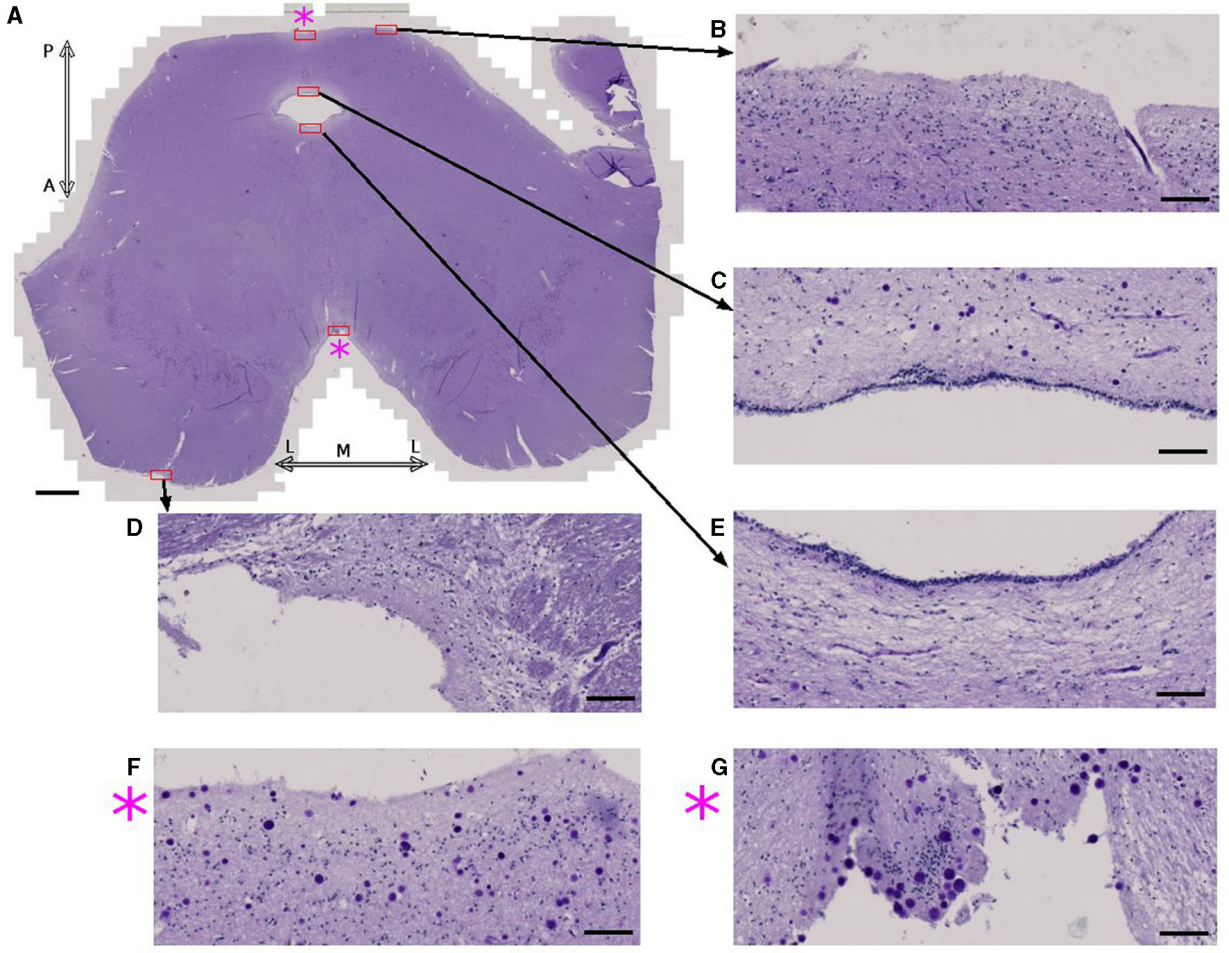
Corpora amylacea are found in some CSF-brain parenchyma boundaries. **(A)** Axial view of the midbrain of subject 4 stained using periodic-acid Schiff method. **(B–G)** are high power magnifications of the regions marked with red rectangles. **(F)** Zoomed view of the posterior medial subpial region. **(G)** Zoomed view of the anterior medial subpial region. P: posterior, A: anterior, M: medial, L:lateral. Scale bars = **(A)** 2 mm, **(B–G)** 100 μm.

Examining the midbrain in an axial plane (Figure 4A), there are two surfaces where brain parenchyma and CSF are in contact. One is the subpial region consisting of the outer surface of the tissue and the other is the ependymal region of the cerebral aqueduct. CA were seldom found in the pial surfaces or ependymal regions (Figures 4B–E) except for the medial region of both the posterior (Figure 4F) and anterior sides (Figure 4G). Results for other samples are shown in Supplementary Figures 8–10. Overall, this study shows that the vicinity of CSF is not the only determinant factor for the accumulation of CA.

### 3.4. Corpora amylacea and blood vessels

There has been contradicting claims on the relationship between blood vessels and CA. Navarro et al. (2018) found blood vessels with several CA distributed along the vessel trajectory. However, Xu et al. (2021) reported that CA were rarely located within perivascular spaces and are more densely populated in avascular zones.

**Figure 5:**
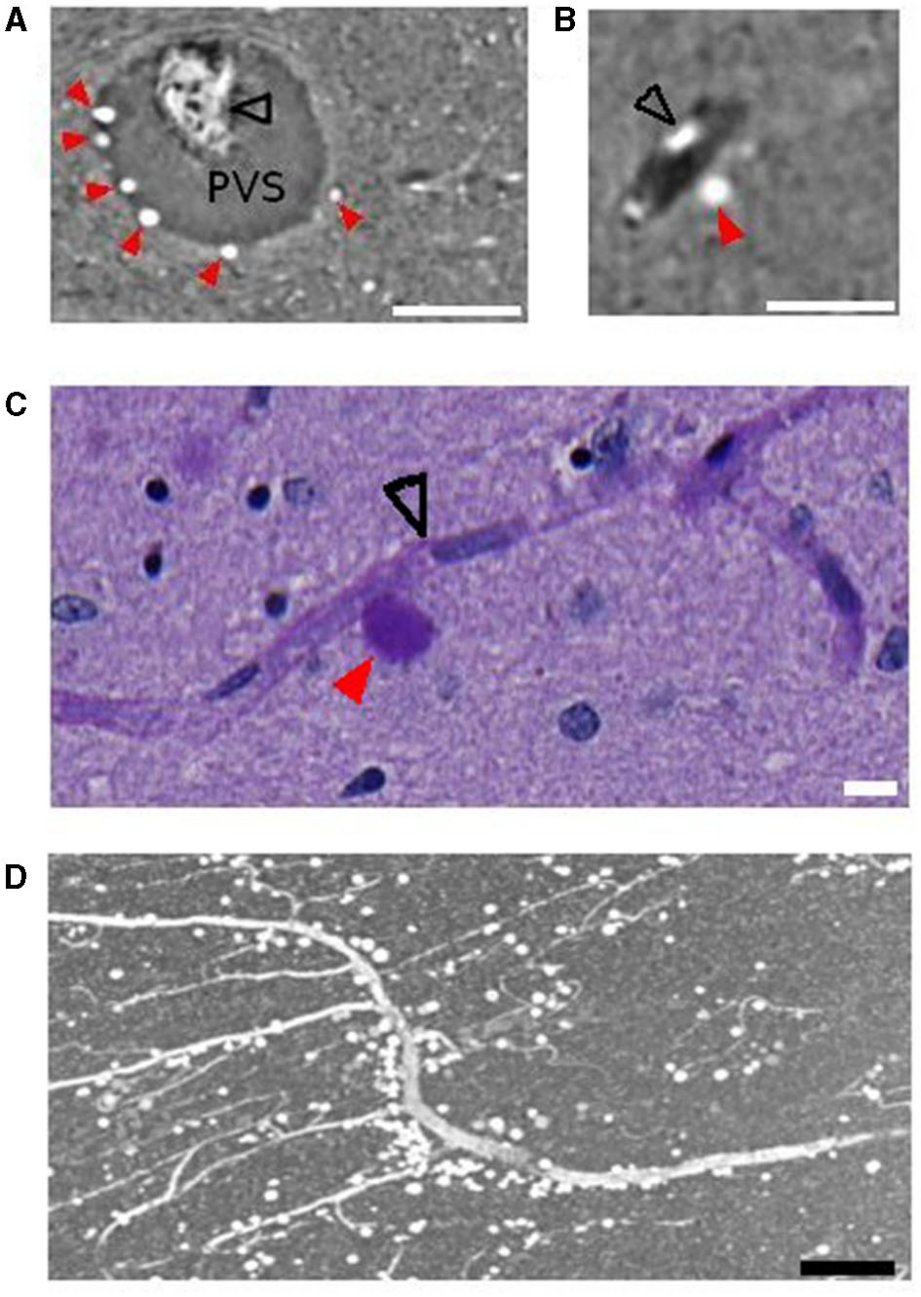
Vessels and corpora amylacea. **(A)** Several corpora amylacea are located within the perivascular space of the central collicular vein. **(B)** A corpus amylaceum is located at the edge of the vessel-tissue boundary. **(C)** A corpora amylacea is in contact with a capillary vessel. **(D)** Anterior medial vessel surrounded by corpora amylacea. Scale bars are 100, 50, 10, 150 μm, respectively, for **(A–D)**. Images shown were obtained with PhC-μCT **(A, B, D)** and microscopy **(C)**. Black open arrows indicate collapsed blood vessel and red filled arrows indicate corpora amylacea. PVS, perivascular space.

In order to investigate the co-localization of CA and blood vessels, we developed a segmentation method that separates blood vessels from CA located in the blood vessel boundaries (Supplementary Figure 3). The resulting vessel structure was then compared with the CA distribution. We found that, overall, 2.2 ± 1.6 % of CA are located in direct contact with the vascular region. The size of the vessel was not a determinant factor for CA since they were found both close to big (Figures 5A, B) and capillary vessels (Figure 5C).

Several CA were observed in the anterior medial vessel in all four subjects. Figure 5D shows a vessel from this region in a sagittal section from subject 4. The vessel is surrounded by CA throughout a long trajectory. High resolution PhC-μCT in this region was only measured from subject 4, so we used microscope images for investigating the anterior medial vessel in the other three subjects (Supplementary Figures 11–15).

### 3.5. Corpora amylacea in magnetic resonance imaging

**Figure 6:**
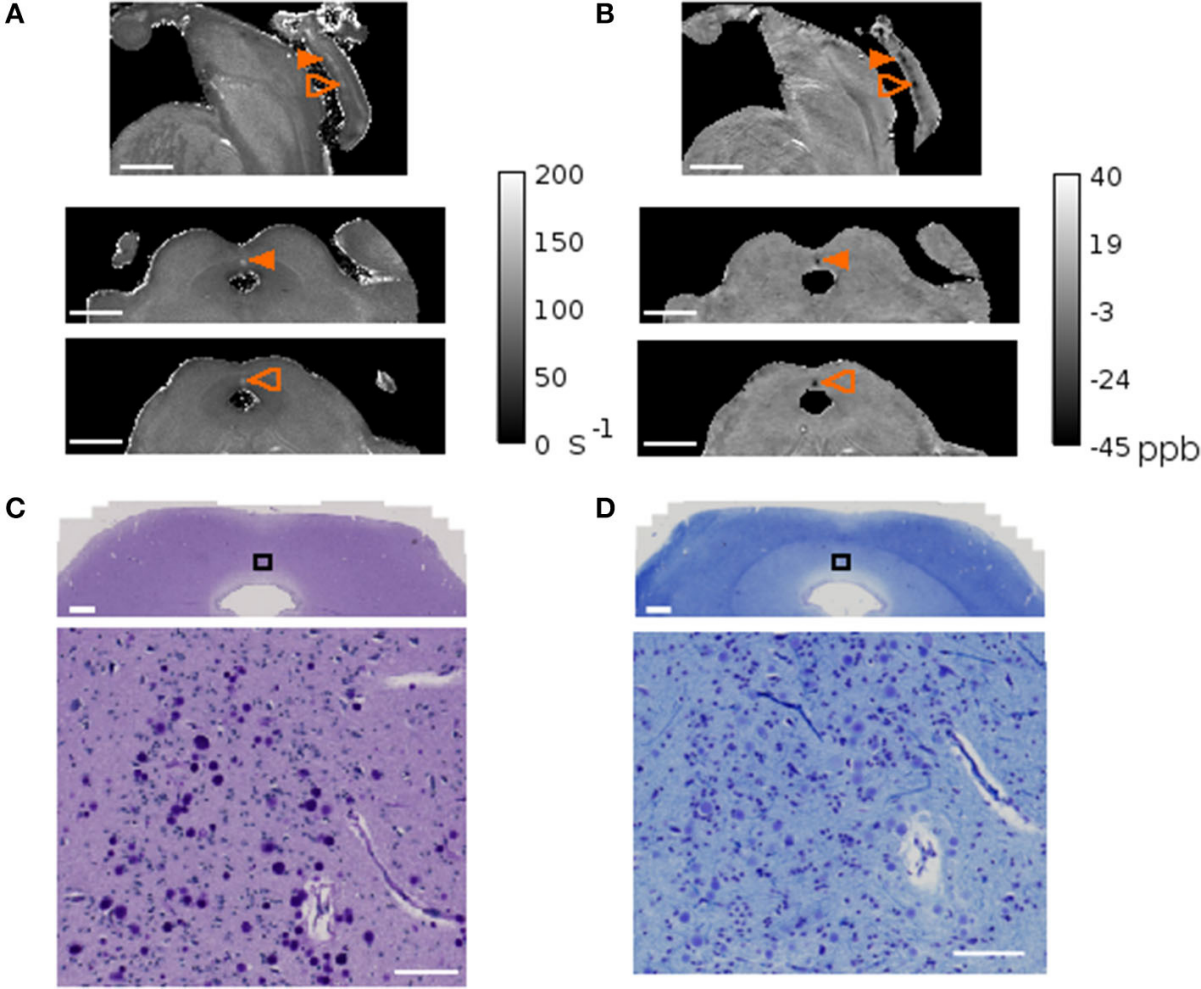
MRI and corpora amylacea. Quantitative map of the transversal relaxation time, R2*(A) and quantitative susceptibility map **(B)** of subject 4. The sagittal view is shown on the top row and the axial views of the planes indicated with orange arrows are shown on the bottom two rows. The arrow indicates the region with high corpora amylacea density characterized by high R2* values and a diamagnetic shift in QSM. Matching region stained with periodic acid Schiff method **(C)** and Luxol fast blue-cresyl violet **(D)** with zoomed inset of black rectangle shown in the bottom panel. Scale bars = **(A, B)** 0.5 mm, **(C, D)** 1mm in top panel, 100 μm in bottom panel.

In conventional MRI, it is unlikely that one voxel contains only CA unless there is a high CA density and the voxel-size is sufficiently small. In the present study we used voxel sizes with sub-millimeter length, leveraging on the high-magnetic field available for preclinical research. In subject 4, which showed exceptionally high CA density in dmPAG, we could observe high R2^*^ values and a diamagnetic shift in quantitative susceptibility maps concomitant with the CA distribution in this region (Figure 6). In the remaining samples, no such prominent effect was observed.

## 4. Discussions

In the present study, we used SR PhC-μCT, MRI, and histology to investigate CA in the human brain stem. Leveraging from automated segmentation, more than 200,000 granules were detected in high-resolution SR PhC-μCT from four subjects (mean age = 75.75). Periodic acid Schiff reaction was used to validate these granules as CA. There are three novel findings in regard to the distribution of CA in the present work: (1) dmPAG accumulates high numbers of CA, (2) not all pial surfaces accumulate CA in the midbrain, and (3) only 2 percent of the CA are located in immediate proximity to the vessels and the perivascular space. Furthermore, in one of the samples we found indications that CA can cause prominent local field inhomogeneities in MRI which lead to alterations in gradient-echo images and quantitative maps. Below, these findings are discussed in more detail.

We investigated the brain stem of four subjects and found the dmPAG to accumulate large numbers of CA. In two of our specimens, we could investigate additional brain regions more caudally (Supplementary Figure 7). We checked a recently released open-source histology dataset (Roetzer-Pejrimovsky et al., 2022a,b) and observed that the distribution of CA was similar to our finding (identification numbers: 38, 361, 1,017, 2,613, 1,874). One hypothesis to explain the accumulation of corpora amylacea in the medial posterior region of the midbrain is the interstitial fluid flow. The colliculi are vascularized with both arteries and veins but the inter-collicular region, where the dmPAG is located, is dominated by large veins that drain to the superior median collicular vein (Duvernoy, 1978). CA accumulation along the midline (Supplementary Figure 4) may result from the interstitial fluid flow toward the midline driven by arterial pulsation in the colliculi. Interstitial fluid flow is an area of active research and further investigation is needed to validate this hypothesis (Hladky and Barrand, 2022).

Prior studies report observing abundant CA in the vicinity of CSF-filled spaces, such as the ependymal or subpial regions (Alder, 1953; Sakai et al., 1969). In the forebrain, large numbers of CA were found in the ependymal region of lateral ventricles (Nam et al., 2012; Xu et al., 2021). However, we did not find this to be true for the cerebral aqueduct. Possibly, the high velocity of CSF flow in the aqueduct (Linninger et al., 2005) may prevent accumulation of the CA granules. For the subpial regions of the midbrain, there were inconsistent findings. In the medial region, both in the posterior and anterior part, there were many CA close to the pial surface. But in other regions, such as the frontopontine and pyramidal tracts, they were seldom found close to the pial surface. The complex geometry of the brain stem can cause microflow in the surrounding CSF (Tangen et al., 2015). In order to associate CSF flow with CA accumulation in selective subpial regions, more in-depth understanding of local CSF-flow would be required.

There have been repeated reports observing CA close to blood vessels (Bakic and Jovanović, 2017; Navarro et al., 2018). Although we found that these CA make up only 2 percent of its entire population in our brain stem samples, it is worth noting that certain vessels tend to accumulate CA throughout a long trajectory (Figure 5D, Supplementary Figures 12–16). Riba et al. (2022) recently suggested the accumulation of CA as a sign for failure the of glymphatic system to remove waste. According to this theory, accumulation of CA should occur predominantly in perivenous spaces rather than in periarterial spaces. The bias of CA distribution toward perivenous compared to periarterial spaces has not been systematically investigated. However, finding the right labels, such as antibodies for identifying vessel type (artery or vein) in *postmortem* tissue, remains a challenge. Furthermore, the contribution of the intramural periarterial drainage pathway should also be assessed as a potential clearance process (Agarwal and Carare, 2020).

Corpora amylacea have been investigated previously using X-ray microanalysis (Singhrao et al., 1993; Tokutake et al., 1995). This technique can identify tissue components like iron, calcium, copper, phosphorus, sulfate, and chloride. Local alterations of the magnetic field caused by small tissue inclusions, like CA, can potentially be detected with gradient-echo MRI techniques. In one of our samples, the CA density was particularly high in dmPAG. In this subject we found concomitant changes in MRI showing higher R2^*^ values and a diamagnetic shift in QSM with respect to the surrounding tissue. The diamagnetic effect thus renders the presence of paramagnetic substances like iron or copper (II), previously reported in Wilson's disease, implausible. Other content like calcium is more likely but would require further analysis to be ascertained. A diamagnetic shift in MRI can also occur in myelin-rich areas, where CA also can be found. Dependent on the magnetic properties of the surroundings, it may thus be difficult to distinguish between MRI signal changes driven by CA, that are much smaller than the voxel size used, and other tissue components. Future studies could take CA isolated from CSF as done in Riba et al. (2019) to obtain more precise information about the magnetic properties and its variability. Such knowledge could be informative for *in vivo* scanning of aging populations.

It is also possible that the components of CA differ in neuropathology. If the current hypothesis of CA serving as a general waste container is true, then CA in patients might contain different components. It has already been shown that molecules observed in CA in Alzheimer patients are different from the normal aging brain (Singhrao et al., 1994; Wander et al., 2022; Riba et al., 2023). In our data, luxol fast blue—cresyl violet staining showed variability of color differentiation in CA (Supplementary Figures 10, 12–15). In subject 1 (68 yr), CA had a much lighter color (Supplementary Figure 12) compared to subject 4 (81 yr), where CA showed a purple color (Supplementary Figure 15). Even within the same slide, the color of CA varied. Cresyl violet is known to stain the Nissl substance in neurons and nuclei of glial cells. Some granules were strongly stained with cresyl violet and some were not. Given such variations, the components of corpora amylacea could perhaps serve as biomarkers for the state of the subject, such as the advancement of the aging process or even pathological states.

Collectively, we show that SR PhC-μCT is a suitable method for understanding the three-dimensional distribution of CA. MRI can be used to obtain additional information regarding their magnetic properties, albeit at a coarser spatial scale. In order to fully understand their distribution in the human nervous system, it will be ideal to obtain high resolution PhC-μCT of the entire brain including cerebrum, cerebellum, and brain stem. The Human Organ Atlas (Walsh et al., 2021) provides hierarchical PhC-μCT data of the whole brain with 25 μm isotropic voxel sizes, occipital lobe with 6 μm, and cerebellum with a 2 μm isotropic voxel size. In PhC-μCT, there is no bleaching effects as in fluorescent imaging and there is no permanent electron beam damage as in electron microscopy. Long exposure-times of fixed brain tissue using synchrotron radiation does not alter tissue contrast for later histological investigation (Saccomano et al., 2018). It should be recognized that there are technical challenges for imaging the whole human brain such as having enough storage space and developing dedicated measurement settings (Stampfl et al., 2023). Nevertheless, such measurements have been done in mice recently (Rodgers, 2022).

In conclusion, we used state of the art biomedical imaging techniques coupled with classical histology methods to study CA within the human brain stem. CA were present throughout the entire midbrain. We found that the dmPAG region of the midbrain had exceptionally high numbers of CA. For the subpial surfaces, not the entire surface but only the anterior and posterior medial parts seem to collect CA. The subependymal region of the aqueduct did not accumulate CA. Further studies should be conducted to understand why CA tend to accumulate in certain regions.

## Data availability statement

The original contributions presented in the study are included in the article/Supplementary material, further inquiries can be directed to the corresponding author.

## Ethics statement

The studies involving humans were approved by Medical Department of the University of Tübingen. The studies were conducted in accordance with the local legislation and institutional requirements. The participants provided their written informed consent to participate in this study.

## Author contributions

JL: investigation, data curation, methodology, software, formal analysis, writing—original draft, and writing—review and editing. AM and UM: resources, investigation, validation, and writing—review and editing. SD: investigation, methodology, data curation, and writing—review and editing. RL: investigation, methodology, and writing—review and editing. GT: investigation, software, and writing—review and editing. TS: resources and writing—review and editing. KS: funding acquisition, data curation, and writing—review and editing. GH: conceptualization, investigation, methodology, software, data curation, formal analysis, supervision, writing—original draft, project administration, writing—review and editing, and funding acquisition. All authors contributed to the article and approved the submitted version.
