# Supplementary material for: Distribution of corpora amylacea in the human midbrain: using synchrotron radiation phase-contrast microtomography, high-field magnetic resonance imaging, and histology
Source: Front Neurosci. 2023 Oct 4;17:1236876. doi: 10.3389/fnins.2023.1236876 (PMC10586329; doi:10.3389/fnins.2023.1236876)
Supplement: Supplementary file 1 [file Data_Sheet_1.PDF]

frontie

Supplementary Material

Distribution of corpora amylacea in the human midbrain : using synchrotron radiation phase-contrast microtomography, high-field magnetic resonance imaging and histology

JY. Lee*, A. F. Mack, U. Mattheus, S. Donato, R. Longo, G. Tromba, T. Shiozawa, K. Scheffler, GE. Hagberg

*** Correspondence:** JY.Lee: ju.young.lee@tuebingen.mpg.de

1 Supplementary Figures

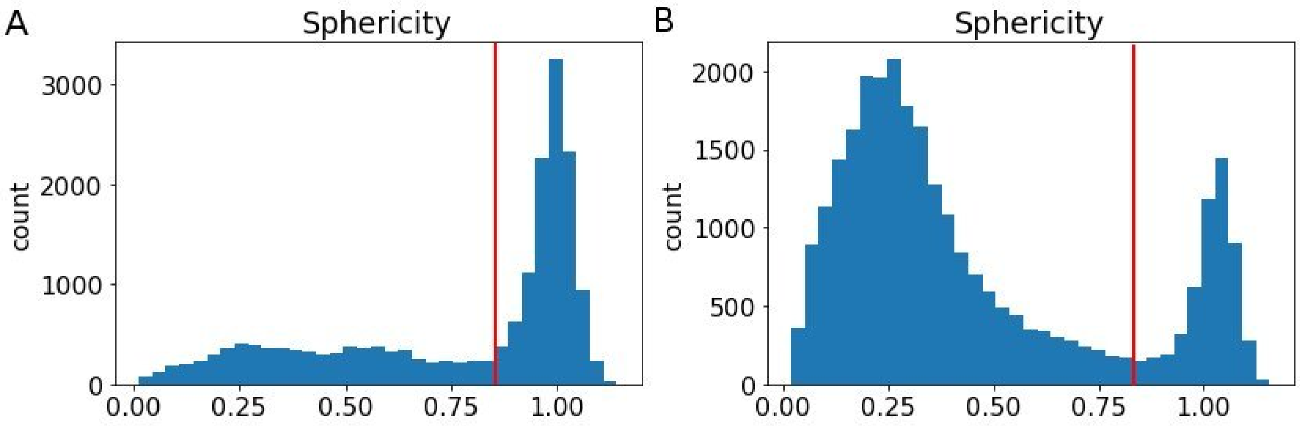

Figure S1. Histogram of sphericity metric. Sphericity of connected components with a volume larger than 55 voxels (365 μm^3) detected in the dorso-medial periaqueductal gray (A) and superior colliculus (B) of subject 4. Since corpora amylacea is known to have spherical shapes, we selected objects with sphericity above 0.85 (indicated with a red vertical line).

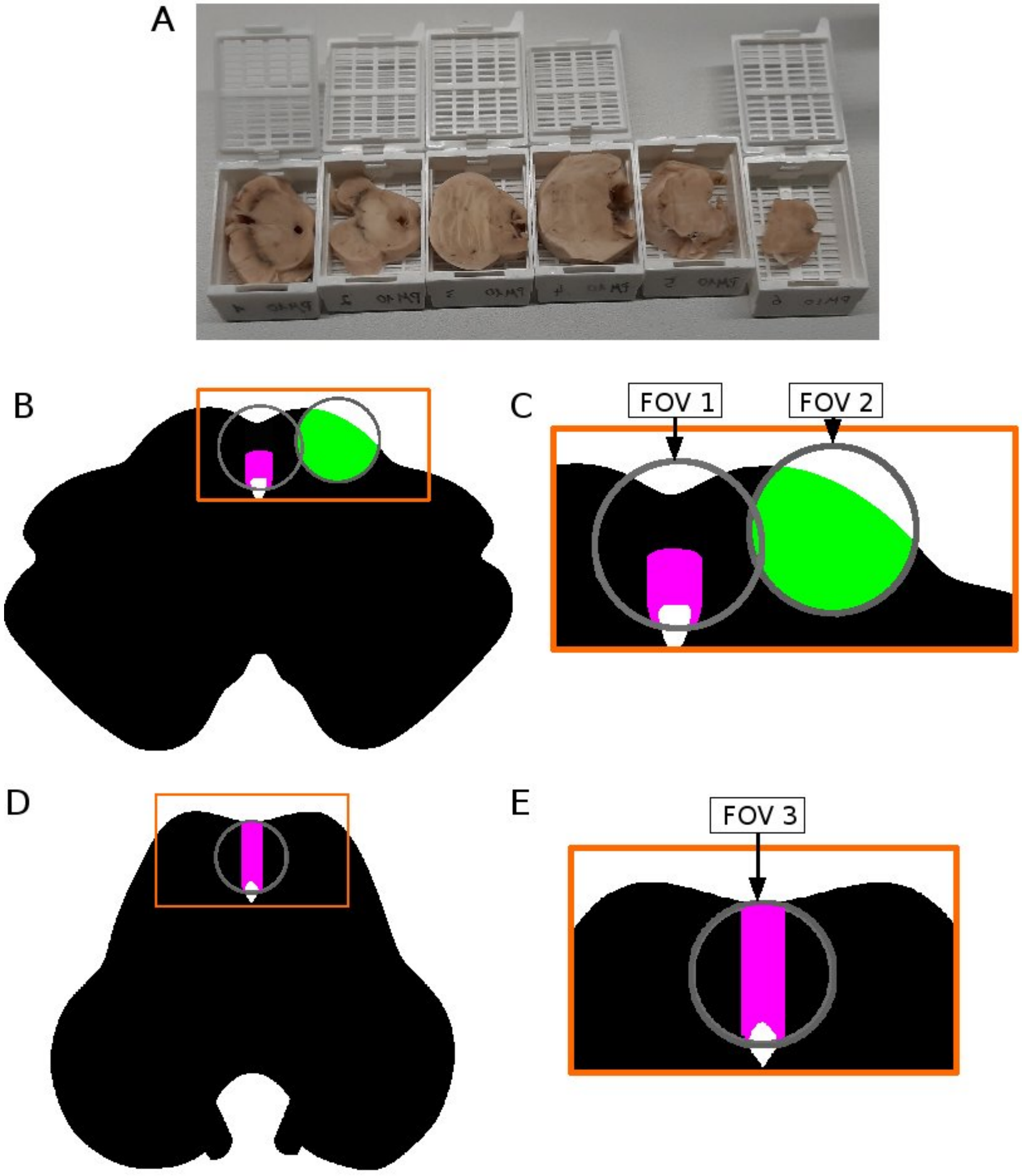

Figure S2. Demonstration of region of interest. (A) Befor paraffin embedding, the brainstem was cut into ca 1cm thick blocks, perpendicular to the length axis of the brainstem. The photograph sample 1 after tissue cutting. The leftmost block includes the superior midbrain including the superior colliculus. The second left block includes the inferior midbrain including the inferior colliculus. The superior midbrain is shown in **(B)** with a zoomed view **(C)**. The inferior midbrain is shown in **(D)** with a zoomed view **(E)**. The gray circles demonstrate the field-of-view covered by the 1 µm SR Ph-µCT scanning. For the region of interest analysis, magenta regions were assigned as dorsomedial periaqueductal gray and the green region as superior colliculus. FOV: field-of-view

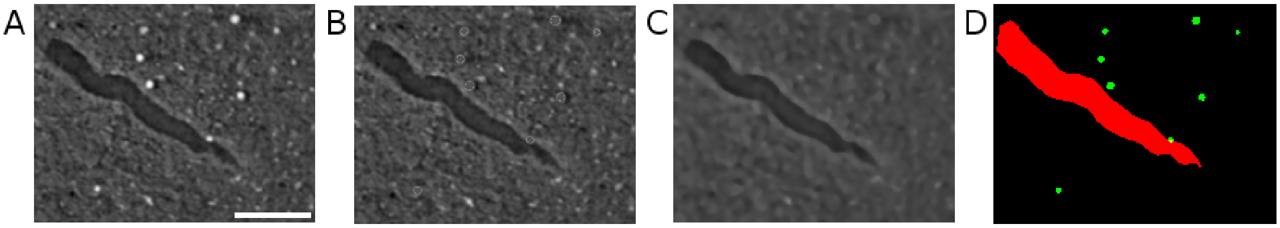

Figure S3 Segmentation pipeline for vessel map and corpora amylacea extraction from SR Ph-µCT images. (A) Original image with a scale bar of 100 µm length. **(B)** Masking corpora amylacea with mode value of the tissue. **(C)** The 3D median filter preserves the vessel-tissue edge while smoothing out circular structures produced in the previous step. The image is further processed using the Derich-Canny edge detection method. **(D)** Final result with vessel map shown in red, corpora amylacea in green.

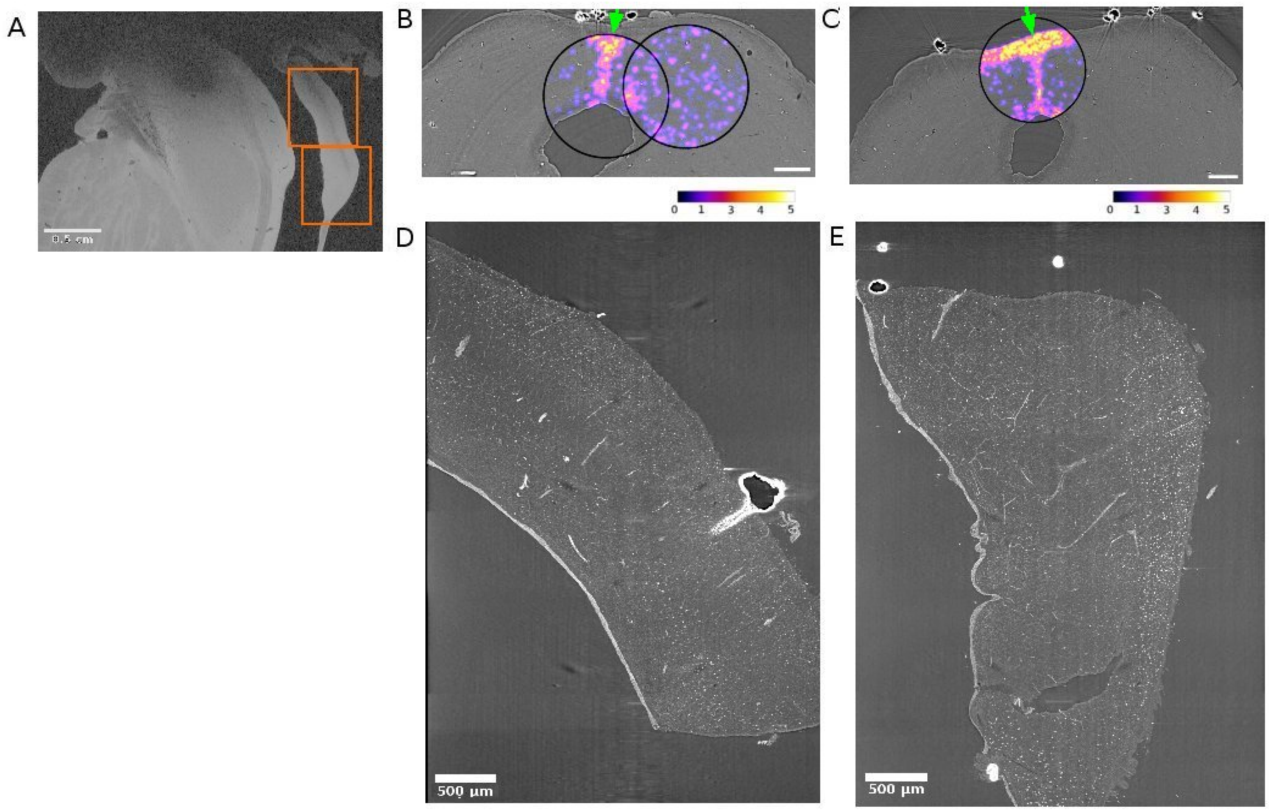

Figure S4. The dorsomedial periaqueductal gray (dmPAG) has abundant corpora amylacea . **(A)** Sagittal view of a T2* weighted MRI of the midbrain. **(B, C)** Axial views of SR PhC-µCT images acquired with 5 µm isotropic voxel size from the midbrain of subject 1. The density of corpora amylacea in the superior (B) and inferior (C) midbrain are shown as density maps obtained from 1 µm isotropic voxel size SR PhC-µCT images. The density of corpora amylacea is clearly higher in the dmPAG region than in the collicular regions. The abundance of corpora amylacea can also be appreciated in the sagittal maximum-intensity projections across the dmPAG at the levels of the superior **(D)** and inferior **(E)** colliculi. The regions used in D and E are indicated with arrows in panels B and C, respectively. The projections are across 60 µm, matching the voxel size in MRI shown in Figure A, where the same regions are indicated with orange rectangles. Scale bars: A = 0.5 cm. B-C = 1 mm, D-E = 0.5 mm. The calibration bar shows the arbitrary unit for density calculation.

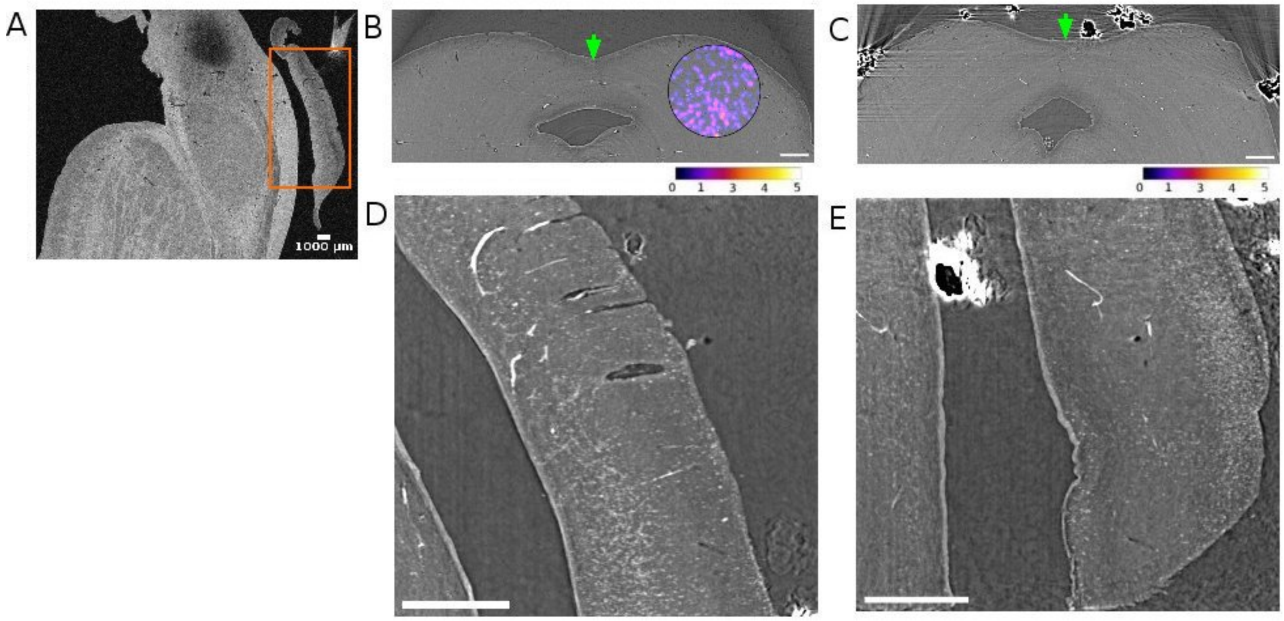

Figure S5. The dorsomedial periaqueductal gray (dmPAG) has abundant corpora amylacea. **(A)** Sagittal view of a T2* weighted MRI of the midbrain. **(B, C)** Axial views of SR PhC-µCT images acquired with 5 µm isotropic voxel size from the midbrain of subject 2. Except for the superior colliculi, 1 µm isotropic voxel size images were not available. Some corpora amylacea can be appreciated in the sagittal maximum-intensity projections across the dmPAG at the levels of the superior **(D)** and inferior **(E)** colliculi. The spatial position of the hyperintensity in these views shows similarity with the hypointense regions in the MRI image. Scale bars = (A) 1 mm, (B-E) 500 µm

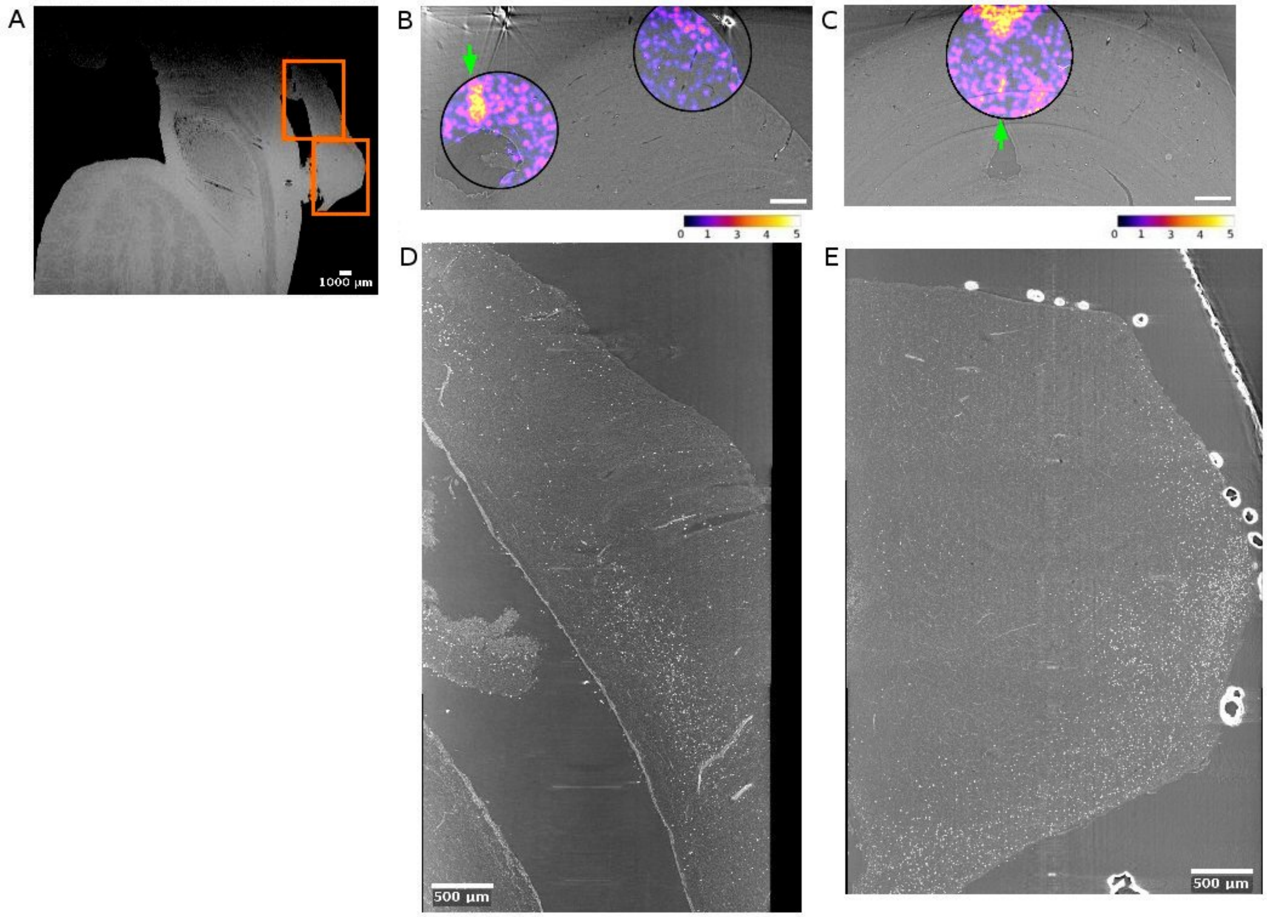

Figure S6. The dorsomedial periaqueductal gray (dmPAG) has abundant corpora amylacea. (A) Sagittal view of a T2* weighted MRI of the midbrain. **(B, C)** Axial views of SR PhC-µCT images acquired with 5 µm isotropic voxel size from the midbrain of subject 3. The density of corpora amylacea in the superior (B) and inferior (C) midbrain are shown as violet/yellow maps. The density maps were obtained from 1 µm isotropic voxel size SR PhC-µCT images. The density of corpora amylacea is clearly higher in the dmPAG region than in the collicular regions. The abundance of corpora amylacea can also be appreciated in the sagittal maximum-intensity projections across the dmPAG at the levels of the superior **(D)** and inferior **(E)** colliculi. The regions used in D and E are indicated with arrows in panels B and C, respectively. The projections are across 60 µm, matching the voxel size in MRI shown in Figure A, where the same regions are indicated with orange rectangles. Scale bars: A = 0.5 cm. B-C = 1 mm, D-E = 0.5 mm. The calibration bar shows the arbitrary unit for density calculation.

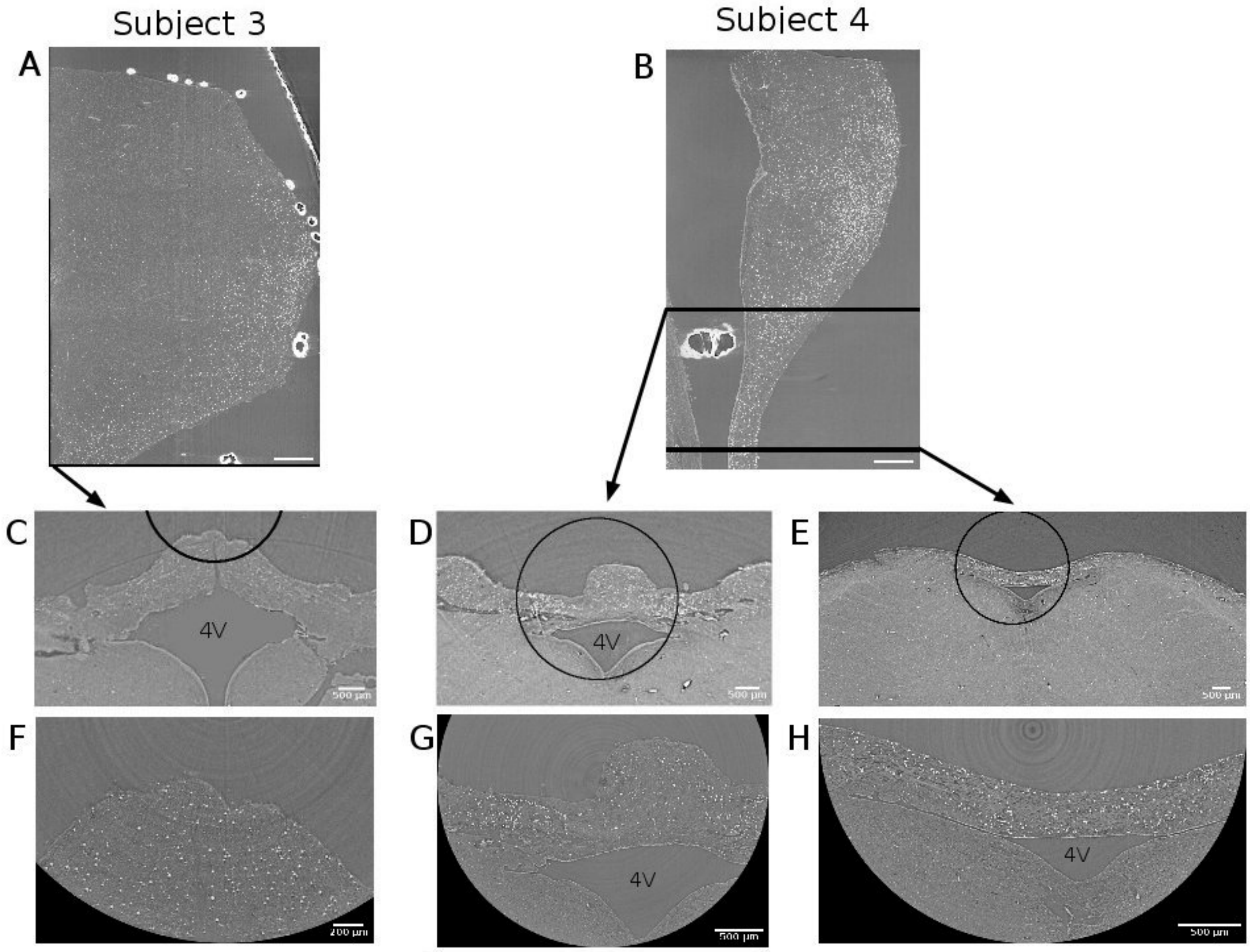

Figure S7. Corpora amylacea are abundant in the transition zone from the inferior collicular commissure to the superior medullary velum. (A-B) Sagittal views of subject 3 and subject 4 are identical to Figure S6E and Figure E2. The black lines indicate the planes shown in C-H. Axial views of PhC-µCT at the level of the fourth ventricle, acquired with 5 µm isotropic voxel size **(C-E)** and 1 µm isotropic voxel size **(F-H)**. 4V: fourth ventricle. Scale bars = 500 µm

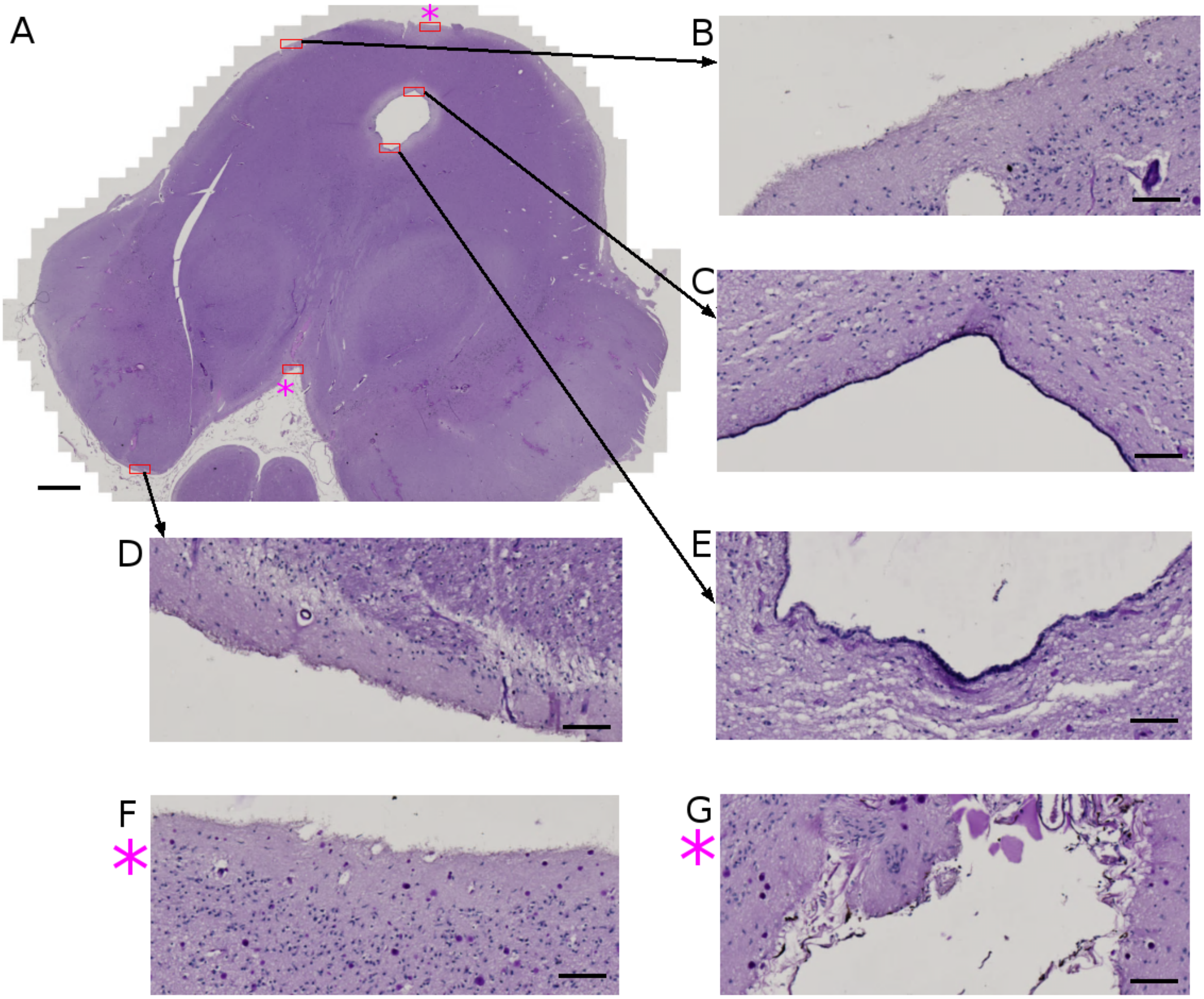

Figure S8. Corpora amylacea are found in subject 1. (A) Axial view of the midbrain of subject 4 stained using periodic-acid Schiff method. (B-G) are zoomed views of the regions marked with red rectangles. (F). Zoomed view of the posterior medial subpial region. (G) Zoomed view of the anterior medial subpial region. Scale bars: 2 mm (A), 100 µm (B-G)

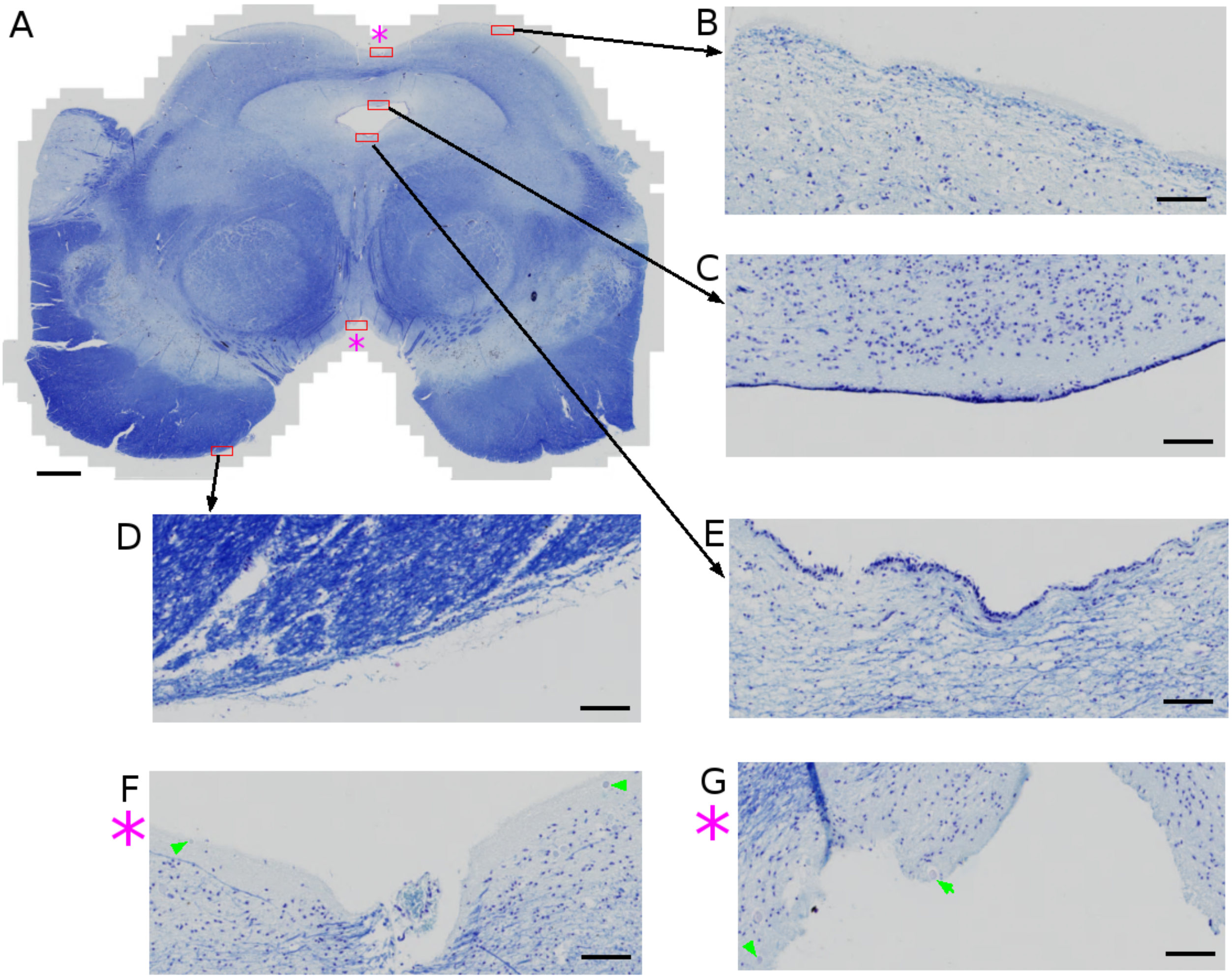

Figure S9. Corpora amylacea are found in subject 2. (A). Axial view of the midbrain of subject 2 stained using Luxol fast blue–cresyl violet method. (B-G) are zoomed views of the regions marked with red rectangles. (F). Zoomed view of the posterior medial subpial region. (G). Zoomed view of the anterior medial subpial region. Scale bars: 2 mm (A), 100 μm (B-G)

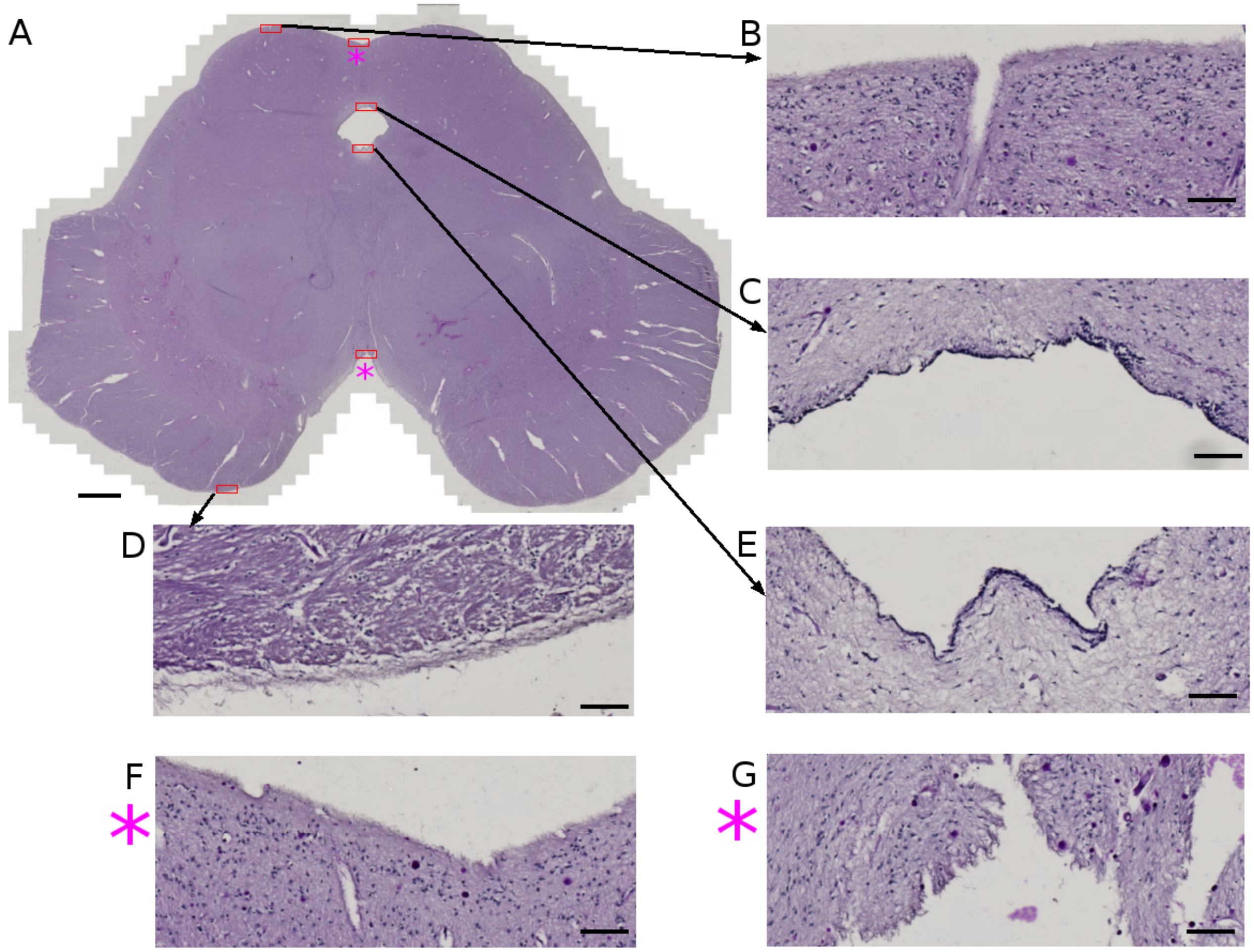

Figure S10. Corpora amylacea are found in subject 3. (A) Axial view of the midbrain of subject 3 stained using periodic-acid Schiff method. (B-G) are zoomed views of the regions marked with red rectangles. (F). Zoomed view of the posterior medial subpial region. (G) Zoomed view of the anterior medial subpial region. Scale bars: 2 mm (A), 100 μm (B-G)

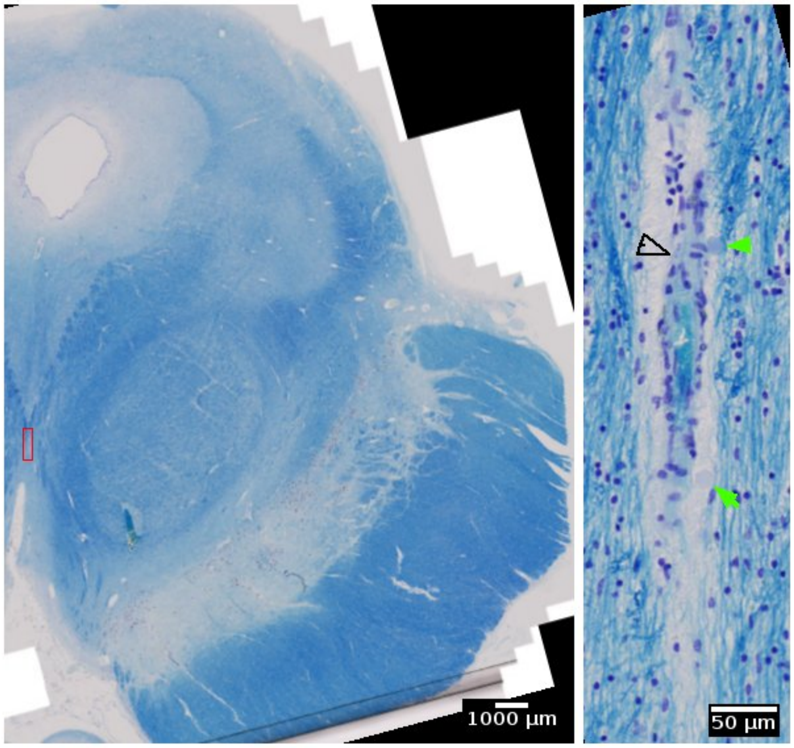

Figure S11. Anterior medial vessel with corpora amylacea in Subject 1. Axial view of the midbrain of subject 1 stained using Luxol fast blue–cresyl violet method. Zoomed view of the anterior medial vessel is shown on the right panel. Green filled arrows indicate corpora amylacea and the black open arrow indicate blood vessel.

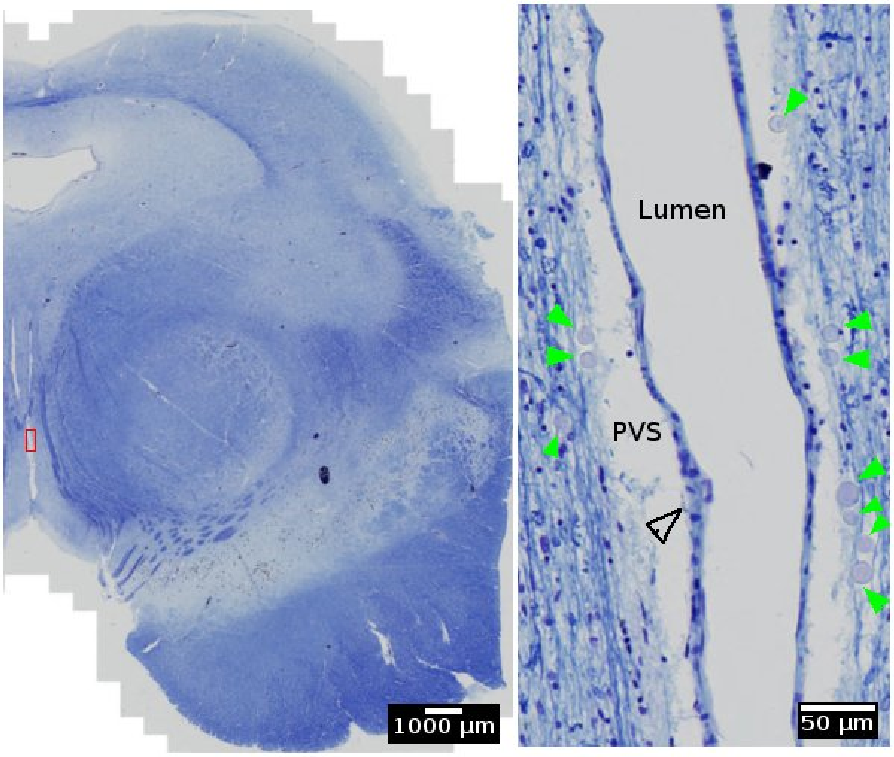

Figure S12. Anterior medial vessel with corpora amylacea in Subject 2. Axial view of the midbrain of subject 1 stained using Luxol fast blue–cresyl violet method. Zoomed view of the anterior medial vessel is shown on the right panel. Green filled arrows indicate corpora amylacea and black open arrow indicate blood vessel.

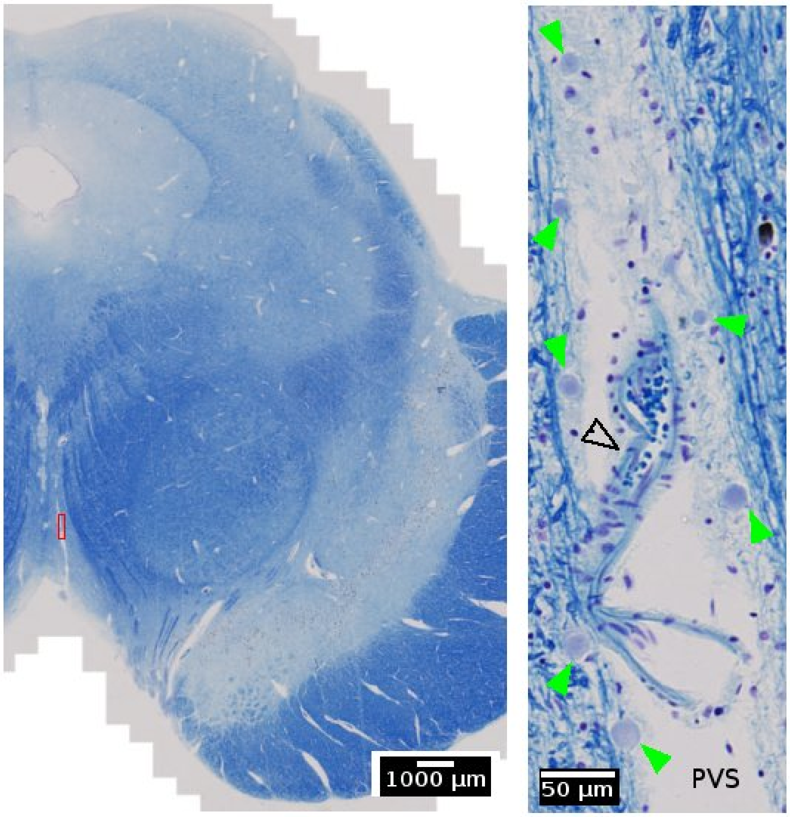

Figure S13. Anterior medial vessel with corpora amylacea in Subject 3. Axial view of the midbrain of subject 1 stained using Luxol fast blue – cresyl violet method. Zoomed view of the anterior medial vessel is shown on the right panel. Green filled arrows indicate corpora amylacea and black open arrow indicate blood vessel. PVS = perivascular space.

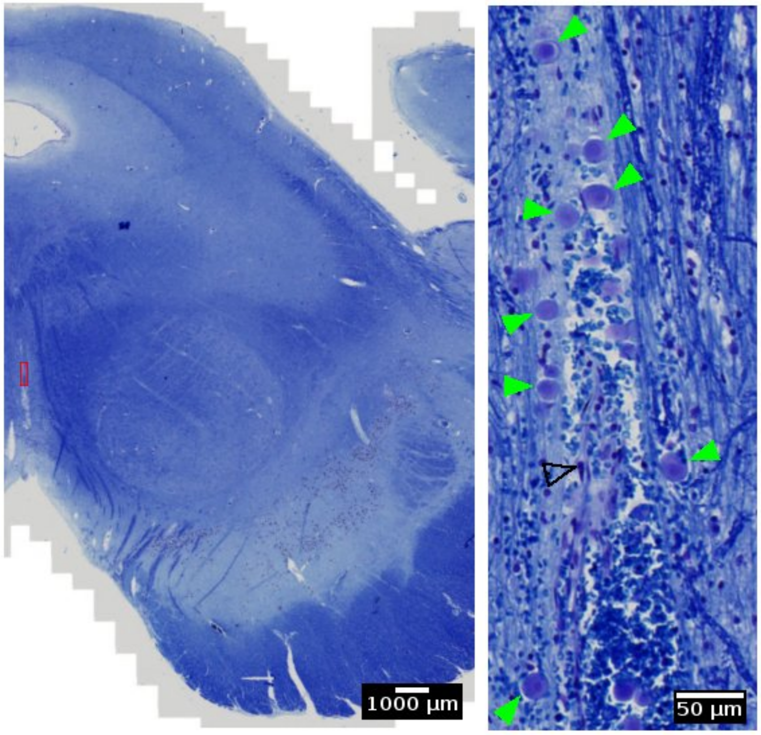

Figure S14. Anterior medial vessel with corpora amylacea in Subject 4. Axial view of the midbrain of subject 1 stained using Luxol fast blue – cresyl violet method. Zoomed view of the anterior medial vessel is shown on the right panel. Green filled arrows indicate corpora amylacea and black open arrow indicate blood vessel.

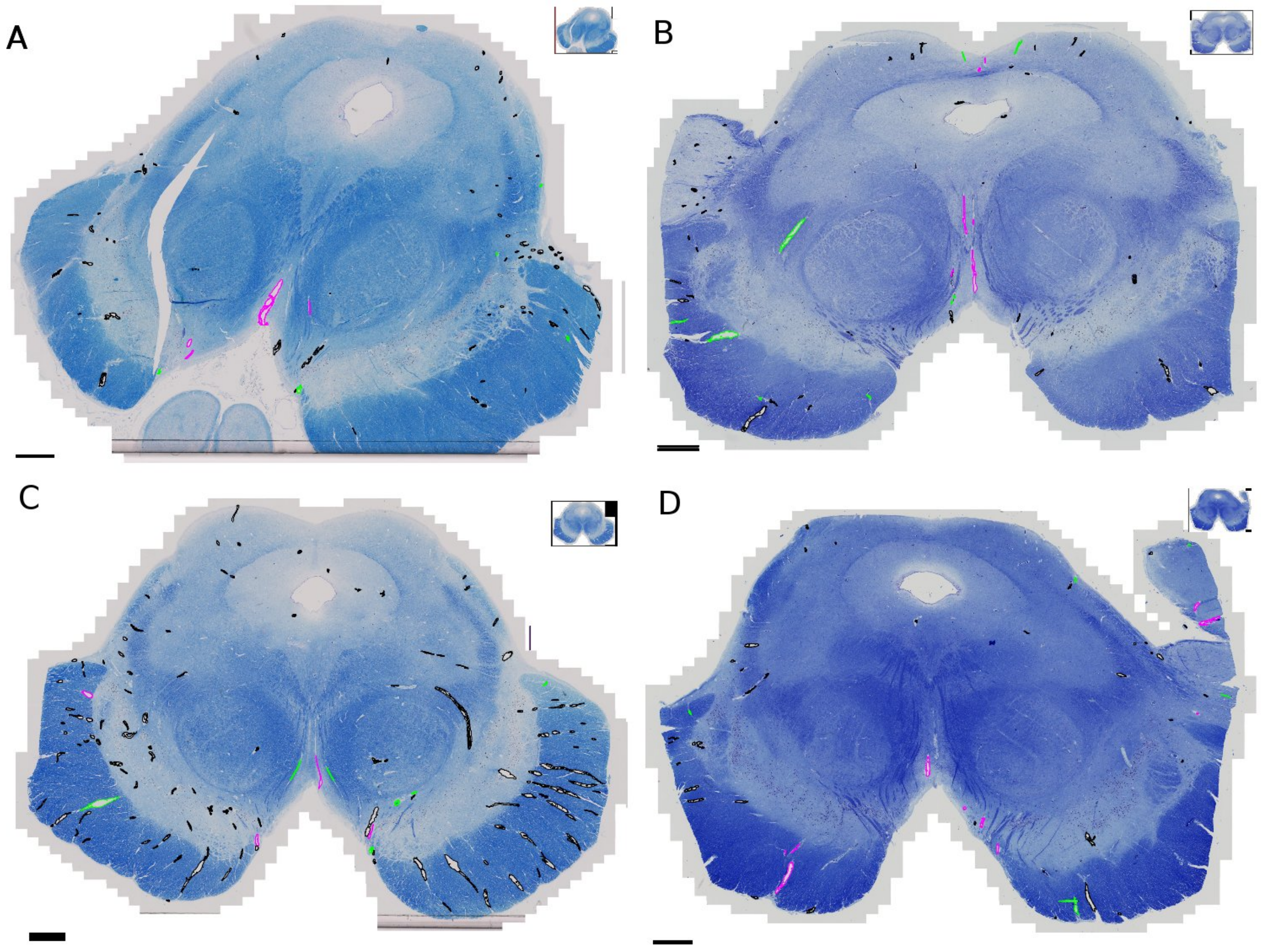

Figure S15. Vessels with corpora amylacea. Each segmented vessel was visually inspected and was labeled with color according to the number of corpora amylacea found in the perivascular spaces. Brown: no corpora amylacea. Green: one corpora amylacea. Magenta: more than two corpora amylacea. Panels A to D are from subjects 1 to 4 in serial order. Scale bars = 2mm

2 Supplementary Tables

	Sample 1	Sample 2	Sample 3	Sample 4
Low Resolution Gradient Echo	Voxel size:<br>200 µm isotropic<br>Echo time:<br>3 : 3 : 18 ms<br>Repetition time:<br>30 ms<br>Flip angle:<br>14°	Voxel size:<br>300 µm isotropic<br>Echo time:<br>2.5 : 2.5 : 15 ms<br>Repetition time:<br>20 ms<br>Flip angle:<br>10°	Voxel size:<br>200 µm isotropic<br>Echo time:<br>3 : 3 : 18 ms<br>Repetition time:<br>30 ms<br>Flip angle:<br>14°	Voxel size:<br>200 µm isotropic<br>Echo time:<br>3 : 3 : 18 ms<br>Repetition time:<br>30 ms<br>Flip angle:<br>14°
High Resolution Gradient Echo	Voxel size:<br>52 x 52 x 94 µm<br>Echo time:<br>6, 14 ms<br>Repetition time:<br>24ms<br>Flip angle:<br>10°	Voxel size:<br>60 µm isotropic<br>Echo time:<br>6.4 ms<br>Repetition time:<br>25 ms<br>Flip angle:<br>10°	Voxel size:<br>52 x 52 x 94 µm<br>Echo time:<br>6 , 14 ms<br>Repetition time:<br>24 ms<br>Flip angle:<br>10°	Voxel size:<br>52 x 52 x 94 µm<br>Echo time:<br>6 , 14 ms<br>Repetition time:<br>24 ms<br>Flip angle:<br>10°

Supplementary Table S1. Parameters for magnetic resonance imaging (MRI). Gradient echo sequence was acquired using 14.1 T MRI (Bruker Biospec). Low resolution gradient echo was used to calculate quantitative maps. The first echoes of high resolution gradient echo are presented in panel A of Figure 2 and Figure S4-6.

Measurement date	2019	2020	2021
Electron energy / current	2.0 GeV / 309 mA	2.0 GeV / 309 mA	2.0 GeV / 249 mA
Details for<br>5 µm isotropic voxel measurements	Measured specimen:<br>Sample 2 block no.1<br>Sample 4 block no.1<br><br>voxel size:<br>4.94 µm isotropic	Not measured	Measured specimen:<br>Sample 1 block no.1, no.2<br>Sample 3 block no.1, no.2<br>Sample 4 block no.2<br><br>voxel size:<br>4.74 µm isotropic
Details for<br>5 µm isotropic voxel measurements	Not measured	Measured specimen:<br>Sample 2 block no.1<br>Sample 4 block no.1<br><br>voxel size:<br>0.94 µm isotropic	Measured specimen:<br>Sample 1 block no.1, no.2<br>Sample 3 block no.1, no.2<br>Sample 4 block no.2<br><br>voxel size:<br>0.94 µm isotropic

Supplementary Table S2. Information on synchrotron radiation phase-contrast microtomography measurement from different beam time schedules. For samples 1, 3 and 4, the block no.1 included the midbrain region at the level of the superior colliculus. The block no.2 included the midbrain region at the level of the inferior colliculus. For sample 2, the block no.1 included covered the entire midbrain. The measurements from 2019 and 2021 overlap with data presented in (Lee et al, 2022, DOI: 10.1038/s41598-022-13282-2)

	Number of Corpora amylacea / tissue volume of the region (count / mm3)			
	Subject 1 (68 yr)	Subject 2 (74 yr)	Subject 3 (80 yr)	Subject 4 (81 yr)
FOV 1	Z level 1 : 5475 / 6.03<br>Z level 2 : 5277 / 13.09<br>Z level 3 : 628 / 3.12	Not acquired	Z level 1 : 7863 / 9.54<br>Z level 2 : 6703 / 12.59<br>Z level 3 : 5909 / 10.95	Z level 1 : 21517 / 10.19<br>Z level 2 : 14668 / 12.33<br>Z level 3: 4151/ 7.24
FOV 2	Z level 1 : 1530 / 9.47<br>Z level 2 : not analyzed due to air bubble artifacts<br>Z level 3 : 1341 / 9.61	Z level 1 : 2570 / 12.43<br>Z level 2: 2933 / 12.91	Z level 1 : 3439 / 10.21<br>Z level 2 : 4095 / 11.94	Z level 1 : 2164 / 9.01<br>Z level 2 : 5207 / 15.24<br>Z level 3 : 3905 / 11.49
FOV 3	Z level 1 : 5746 / 7.65<br>Z level 2: 6691/ 10.23	Not acquired	Z level 1 : 4250 / 8.22<br>Z level 2 : 14011 / 17.17	Z level 1 : 21631 / 11.21<br>Z level 2 : 35096 / 10.83<br>Z level 3: 14856 / 5.46
Total	26688	5503	46270	123195

Supplementary Table S3. The number of corpora amylacea segmented. Field-of-view (FOV) number corresponds to the demonstration noted in Supplementary Figure 1. FOV 1 covers the posterior medial midbrain. FOV 2 covers the superior colliculus. FOV 3 covers the posterior medial midbrain of the lower block.

3 Supplementary Video

Supplementary Video 1

The region adjacent to Figure 1A is shown side by side with the original microtomography (left) and segmented corpora amylacea overlayed. (right)
